# Nitrogen deposition and climate change effects on tree species composition and ecosystem services for a forest cohort

**DOI:** 10.1002/ecm.1345

**Published:** 2019-02-05

**Authors:** George Van Houtven, Jennifer Phelan, Christopher Clark, Robert D. Sabo, John Buckley, R. Quinn Thomas, Kevin Horn, Stephen D. LeDuc

**Affiliations:** ^1^ RTI International 3040 Cornwallis Road Research Triangle Park North Carolina 27709 USA; ^2^ Office of Research and Development U.S. Environmental Protection Agency 1200 Pennsylvania Avenue NW Washington D.C. 20460 USA; ^3^ Department of Forest Resources & Environmental Conservation Virginia Tech University 310 West Campus Drive Blacksburg Virginia 24061 USA

**Keywords:** *Acer rubrum*, *Acer saccharum*, carbon sequestration, community composition, critical loads, forests, *Liriodendron tulipifera*, *Prunus serotina*, *Quercus rubra*

## Abstract

The composition of forests in the northeastern United States and the ecosystem services they provide to future generations will depend on several factors. In this paper, we isolate the effects of two environmental drivers, nitrogen (N) deposition and climate (temperature and precipitation) change, through an analysis of a single cohort of 24 dominant tree species. We assembled a tree database using data from U.S. Forest Service Forest Inventory and Analysis monitoring plots. Applying observed species‐specific growth and survival responses, we simulated how forest stands in a 19‐state study area would change from 2005 to 2100 under 12 different future N deposition–climate scenarios. We then estimated implications for three selected forest ecosystem services: merchantable timber, aboveground carbon sequestration, and tree diversity. Total tree biomass (for 24 species combined) was positively associated with both increased N deposition and temperatures; however, due to differences in the direction and magnitude of species‐specific responses, forest composition varied across scenarios. For example, red maple (*Acer rubrum*) trees gained biomass under scenarios with more N deposition and more climate change, whereas biomass of yellow birch (*Betula alleghaniensis*) and red pine (*Pinus resinosa*) was negatively affected. Projections for ecosystem services also varied across scenarios. Carbon sequestration, which is positively associated with biomass accumulation, increased with N deposition and increasing climate change. Total timber values also increased with overall biomass; however, scenarios with increasing climate change tended to favor species with lower merchantable value, whereas more N deposition favored species with higher merchantable value. Tree species diversity was projected to decrease with greater changes in climate (warmer temperatures), especially in the northwestern, central, and southeastern portions of the study area. In contrast, the effects of N deposition on diversity varied greatly in magnitude and direction across the study area. This study highlights species‐specific and regional effects of N deposition and climate change in northeastern U.S. forests, which can inform management decision for air quality and forests in the region, as well as climate policy. It also provides a foundation for future studies that may incorporate other important factors such as multiple cohorts, sulfur deposition, insects, and diseases.

## Introduction

Growth in human populations and economic activity over the last century have contributed to large increases in emissions of nitrogen (N) compounds and greenhouse gases (GHG) to the atmosphere. As a result, N deposition from anthropogenic sources across the globe has increased by more almost threefold over historical levels (Fowler 2013, Kanakidou et al. 2016). And although N deposition in the eastern United States has decreased since the early 1990s (Beachley et al. 2016), it remains 2–10 times above pre‐settlement levels. In addition, average global surface temperatures have steadily risen since the early 1900s (Cramer et al. 2014, IPCC 2014). Both individually and interactively, these stressors have had wide ranging impacts on the structure and functioning of terrestrial ecosystems, including alterations in species distribution and diversity of trees within forested ecosystems (Parmesan 2006, Collins and Larry 2007). Forested lands account for almost a third of the total land area in the United States (U.S.) and contain over 800 different tree species (Oswalt et al. 2014). Inevitably, changes in the relative abundance of different tree species within these forests can impact the benefits that humans receive, both directly and indirectly, from these natural systems (Clark et al. 2017, Irvine et al. 2017).

Elevated N deposition can have both nutrient enrichment and acidifying impacts, and the resulting effects on trees within forest ecosystems can be both positive and negative. These effects can occur through direct impacts on foliage and through alterations in soil chemistry (Bobbink 2010, Bobbink and Hettelingh 2011, Pardo 2011). As a nutrient, N availability limits the growth of many forested systems (Elser et al. 2007, LeBauer and Treseder 2008). Consequently, increased N deposition can often increase tree growth. However, because different tree species have different sensitivities (in both magnitude and direction) to elevated N (Magill et al. 2000, McNulty et al. 2005, Thomas et al. 2010, Dietze and Moorcroft 2011, Lovett and Goodale 2011), higher levels of N deposition can lead to changes from the historical species composition of forests over time. In addition, N deposition can cause less competitive species in the understory (including tree saplings) to be excluded from the community and/or cause invasive and fast‐growing species to increase in abundance (Bobbink 2010, Porter et al. 2013). Acting as an acidifying agent, N can also directly damage foliage, leach nutrient base cations from the soil, and increase the solubility of aluminum (Al), which is phytotoxic to some tree species (Eldhuset et al. 1987, Godbold et al. 1988, De Wit et al. 2001, Yang et al. 2015). Furthermore, this varying sensitivity of trees to increased N and acidified soil conditions can interact with co‐occurring stressor, such as pest damage, disease, fire, and tropospheric ozone, further negatively impacting the health of trees and altering forest composition (Strengbom et al. 2002, 2003, Nordin et al. 2006, Yamaguchi et al. 2007, Marzuoli et al. 2016).

Climatic conditions can also influence tree species composition and diversity. Altered precipitation and temperature patterns can alter growing season conditions and length, thereby altering the location of optimal habitats (Walther et al. 2002, Parmesan and Yohe 2003, Root et al. 2003, Walther 2003, Parmesan 2006). They can also result in changes to the timing of episodic climatic disturbance events, such as drought and frost, and contribute to evapotranspiration and water stress (Dale 2001). Both elevated N deposition and climate stresses often lead to increased damage and/or mortality of sensitive tree species (McNulty and Boggs 2010, Thomas et al. 2010, Dietze and Moorcroft 2011).

The importance of understanding the impacts of N deposition and climate on forests is underscored by the vital and diverse role that forest ecosystems play as a natural asset that can provide many different ecosystem services to human populations (Ninan and Inoue 2013, Binder et al. 2017). Forests offer “provisioning services” by providing a wide variety of natural products that are directly used and often extracted by humans to provide sustenance, protection, and health. Forests also offer “cultural” services by providing natural environments that humans value for recreational, aesthetic, spiritual, educational, and other uses, and they provide “regulating” services through their role in natural processes that control and maintain the quantity and quality of air, water, climate, wildlife, and soil‐based resources. The level and types of ecosystem services provided by these assets depend, in many ways, on the types and distribution of tree species contained within these natural systems. Forests also provide “supporting services” such as nutrient cycling, which are needed for the production of all other ecosystem services (Millennium Ecosystem Assessment 2005).

To advance understanding of these impacts, this study had two main objectives. The first objective was to project changes in forest composition in response to future changes in N deposition and climate conditions by using previously derived empirical relationships between these factors and the growth and survival of individual tree species (Thomas et al. 2010). Using these species‐specific response estimates, we modeled changes in the composition of a single mixed‐age cohort of trees from 2005 to 2100 under alternative deposition–climate futures. Because Thomas et al. (2010) found large variation across species in both the direction and magnitude of responses to changing N deposition and climate, we expected to see substantial differences in forest composition under the different scenarios.

The second objective was to estimate how ecosystem services provided by the current cohort of trees may change over the course of the 95‐yr period. Given that climate and N deposition can each have diverse effects on species composition that may be synergistic or antagonistic, it is expected that some services may be augmented by these combined effects while others may diminish. In this study, we focused our evaluation on three commonly cited services provided by forests: provisioning services provided merchantable timber, regulating services provided by C sequestration, and tree diversity, which is a surrogate for many individual services (Schuldt 2010, Yang 2013). Forest tree diversity can be considered as a source of “intermediate” ecosystem services (Landers and Nahlik 2013, Bell et al. 2017), in that tree diversity can influence several “final” ecosystem goods and services that are directly used by people, such as timber volume for commercial use and wild game for hunting (Gamfeldt et al. 2013). Tree diversity itself can also be considered a final ecosystem service for nonconsumptive users who value this diversity for ceremonial or bequeathing purposes (Landers and Nahlik 2013). By addressing these two objectives, this study also provides a blueprint for future assessments analyzing the impacts of global change on forests and the ecosystem services they provide.

Although other studies have modeled the impacts of N deposition (Ollinger et al. 2002) and climate change on forests and the forest industry in the U.S. (Scheller and Mladenoff 2005, Kirilenko and Sedjo 2007, Thompson et al. 2011, Duveneck et al. 2014, Beach 2015), to our knowledge this is the first study that applies species‐specific estimates of N and climate sensitivity to model future changes in forest composition and to assess the effect of these changes on forest ecosystem services.

## Methods

### Tree database

**Figure 1:**
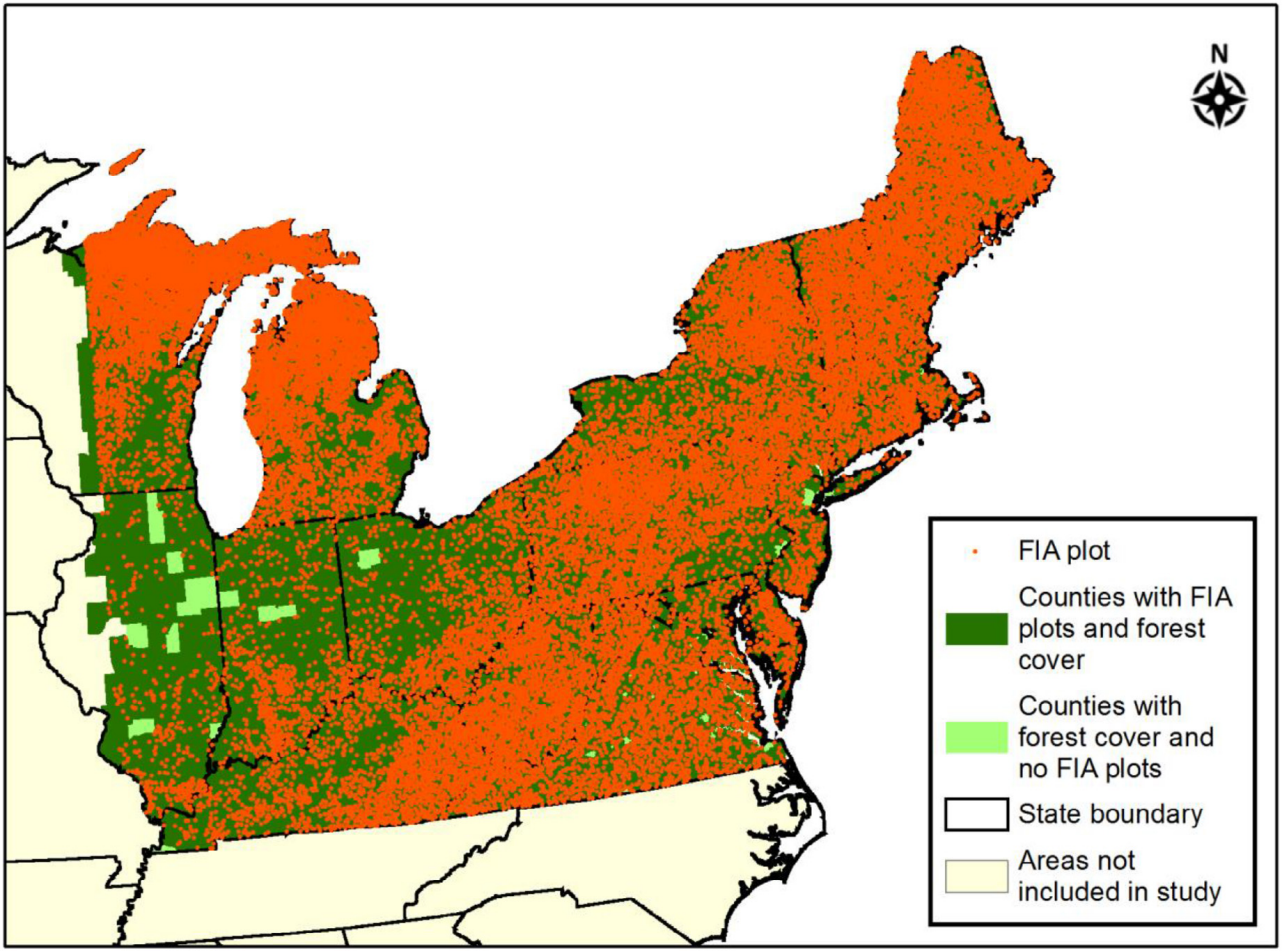
Locations and county representation of the U.S. Forest Service Forest Inventory and Analysis (USFS FIA) plots that were included in the study tree database. Presence of forest cover within each county was based on 2011 National Land Cover (NLCD; https://www.mrlc.gov/nlcd2011.php). “Areas not included in the study” represents area outside of the Thomas et al. (2010) and the current study area.

A tree database was assembled to produce an initial, starting forest cohort of trees for the study. This database consists of tree data from U.S. Forest Service (USFS) Forest Inventory and Analysis (FIA) monitoring plots located in 19‐state study area in the northeastern United States delineated by Thomas et al. (2010) (Fig. 1). The study area was selected for consistency with the tree growth and survival relationships developed by Thomas et al. (2010) and because the northeastern region of the United States is an area with historically high levels of N deposition. Within this area, a total of 26,895 plots were selected for the database that met a series of criteria: (1) had at least two measurements between 2000 and 2011, (2) was not planted (i.e., naturally regenerated), and (3) current stand did not have evidence of harvesting, silvicultural treatment, or significant disturbance (i.e., damage from insects, disease, fire, or extreme weather) since the last measurement. Within these plots, only data for “trees” were included in the database (total of 527,529 trees), where trees are defined as having a stem diameter of >5 inches (12.7 cm), for the 24 species modeled by Thomas et al. (2010). Thus, the trees selected for analysis represented re‐measured forest trees grown under unmanaged and relatively undisturbed conditions. Total biomass (aboveground) of each tree was then estimated using the measured FIA tree diameters and allometric biomass equations used by Jenkins et al. (2003). These species‐specific biomass equations are applicable to all trees with greater than a 2.5 cm diameter. Each tree within the database was treated as “representative” of trees within the forest type throughout the county where the FIA plot was found, and plot‐level total tree counts and individual tree biomass estimates were expanded to the county level using the FIA database tree‐ and plot‐specific expansion factors and equations (O'Connell et al. 2017). In summary, the initial, starting unmanaged forest condition within the tree database (hereafter referred to as the “2005 forest condition” to represent a common starting year for the forest composition model simulations), consisted of a total of ~15 billion trees and 6.3 billion metric tons (MT) of total biomass across the 19‐state area.

### Deposition and climate scenarios

A total of 12 future scenarios were evaluated in this study. These scenarios consisted of all possible combinations of three N deposition and four climate scenarios, which were selected to represent a broad range of potential deposition, temperature, and precipitation futures based on historical trends, U.S. policy, and Intergovernmental Policy on Climate Change (IPCC) scenarios.

### N deposition scenarios

The three N deposition scenarios consisted of annual total (wet plus dry) N deposition estimates from 2005 to 2100 applied to each USFS FIA plot (using publicly available coordinates) in the 19‐state study area. These deposition scenarios included: (1) contemporary (circa 2005) N deposition repeated into the future (hereafter referred to as “Constant”), (2) reductions in N deposition according to U.S. Environmental Protection Agency (EPA) projections under Clean Air Act (CAA) Tier III regulations (hereafter referred to as “CAA2025”) out to the year 2025 and then constant to 2100, and (3) return to pre‐European N deposition (hereafter referred to as “Return to PE”), represented by 0.4 kg N/ha across the entire study region.

**Figure 2:**
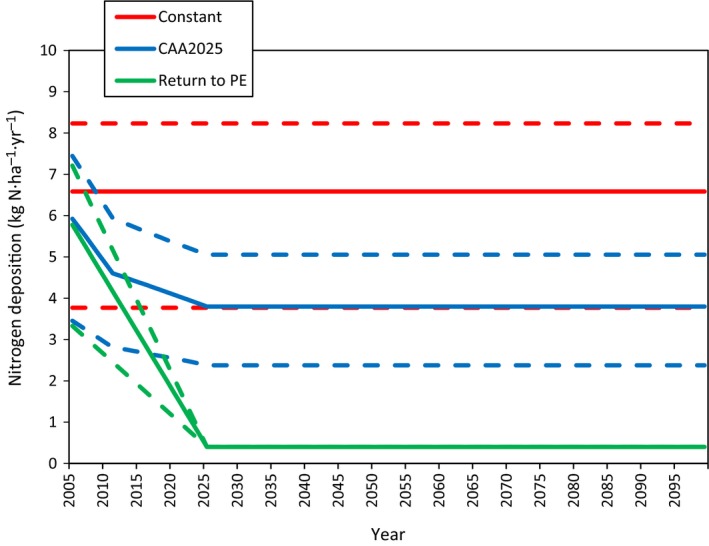
Average annual N deposition from 2005 to 2100, according to the three N deposition scenarios. Solid lines represent the 50th percentile and the dashed lines represent the 5th and 95th percentile of average annual deposition across the study area.

To maintain consistency with the total (wet plus dry) N deposition estimates used to develop the Thomas et al. (2010) growth and survival relationships, all three deposition scenarios in this current study used the 800‐m grid resolution 2000–2004 average National Atmospheric Deposition Program (NADP) wet deposition and the Clean Air Status and Trends Network (CASTNet) dry deposition data set used by Thomas et al. (2010) as the “base” deposition data set. Capturing variation in N deposition within each 800‐m grid, for example, due to variation in elevation, is beyond the scope of this study. Dry deposition estimates from CASTNet are known to be lower than more contemporary deposition estimates such as those from Community Multiscale Air Quality (CMAQ; Bash et al. 2013) or total deposition (TDEP; Schwede and Lear 2014). Therefore, to prevent the over or under estimation of tree growth and survival responses the three deposition scenarios in this current study were determined by percent change scaling factors applied to the Thomas et al. (2010) “baseline” deposition. Fig. 2 presents N deposition from 2005–2100 according to the three deposition scenarios summarized across the study area. The Constant deposition scenario consisted of the 2000–2004 average baseline deposition rate from Thomas et al. (2010) repeated for each year from 2005–2100, representing a period when deposition has declined from peak levels in the 1980s and 1990s, but remained 16.5 times higher than pre‐European settlement levels over the study area. A map of baseline N deposition levels (in 2005) is shown in Appendix S1: Fig. S1. The CAA2025 and Return to PE deposition scenarios both started from this same baseline deposition rate (in 2002 for consistency with the CAA2025 deposition data sets), but then declined until 2025 and remained constant until 2100. The 2025 target year was selected for consistency with the time frame of the CAA2025 deposition estimates.

The CAA2025 deposition scenario and scaling factor were developed using results from version 5.0.2 of the Community Multi‐scale Air Quality (CMAQ) model, which includes bidirectional exchange of NH_3_ (Bash et al. 2013; *available online*).^1^ Declines in N deposition were separately generated for the periods 2005–2011 and 2011–2025, using two different intervals of CMAQ deposition estimates, because a single set of CMAQ estimates was not available for the entire 2005–2025 period. For the first interval (2005–2011), starting from the Thomas et al. (2010) average baseline deposition rate, we applied the average annual percent decline in CMAQ total N deposition between 2002 and 2011 (CMAQ, 2007), and interpolated linearly for 2002–2011. From that point, for the period from 2011 to 2025, we applied the percent decreases in CMAQ total N deposition estimated for the same period (USEPA 2014; Robin Dennis, *personal communication*). For the full period (2005–2025) the median decline in N deposition was 36%. Both sets of CMAQ model estimates used 2011 meteorology in the simulations. For the remainder of the simulation period (2026–2100) for the CAA2025 scenario, N deposition was held constant at the 2025 deposition level.

The Return to PE deposition scenario was developed using an estimate of N deposition (0.4 kg N·ha^−1^·yr^−1^) prior to European settlement and industrial growth, comparable to that reported in other studies (Holland et al. 1999, Galloway 2004, Baron 2006). This scenario therefore consisted of the difference between the Thomas et al. (2010) baseline (2002–2004) and 0.4 kg N·ha^−1^·yr^−1^ applied as a linear decrease in deposition from 2002 to 2025, followed by 2025 deposition (0.4 kg N·ha^−1^·yr^−1^) repeated from 2026 to 2100.

**Figure 3:**
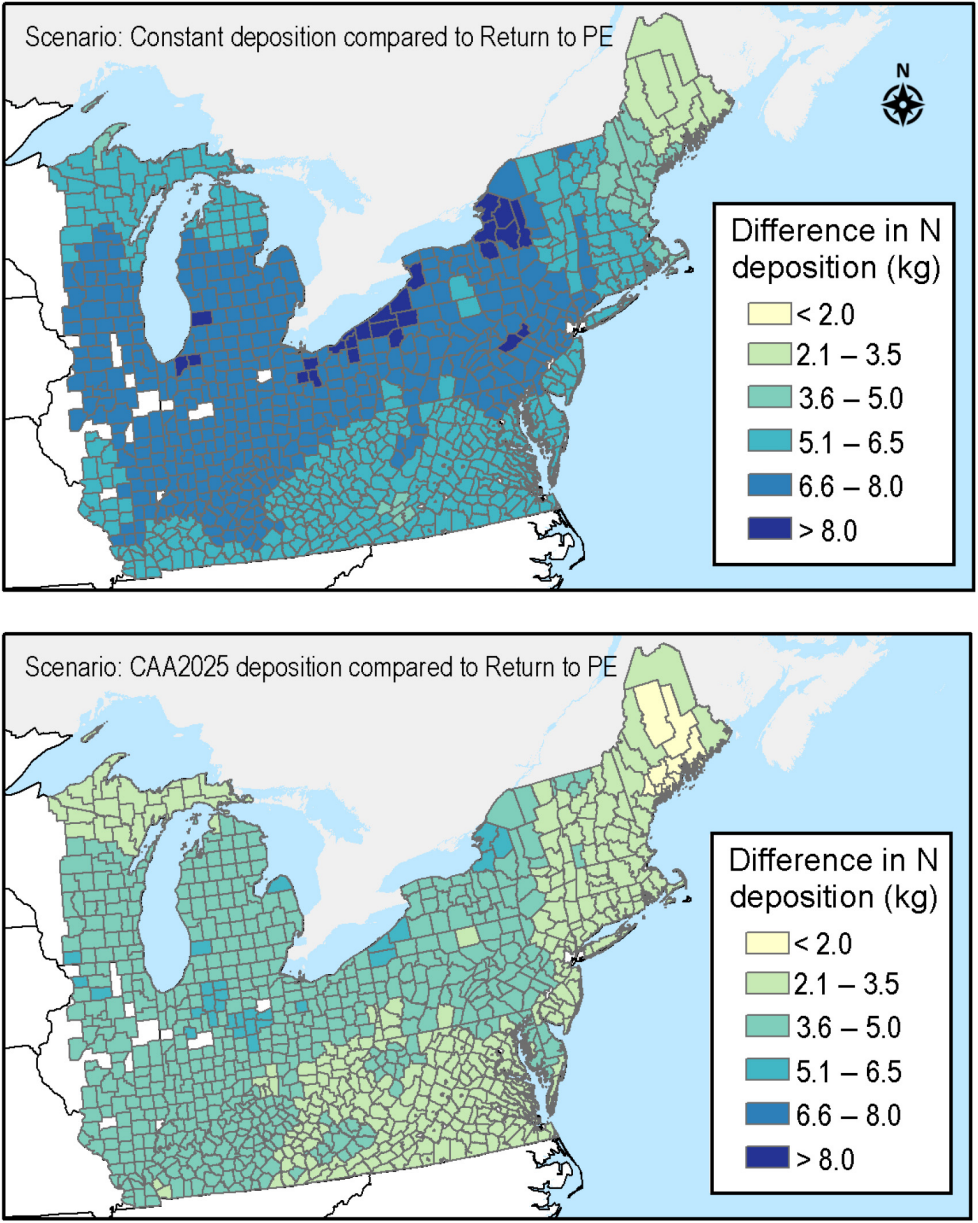
County‐level differences in average annual N deposition, relative to the return to PE scenario (i.e., 0.4 kg N·ha^−1^·yr^−1^ across the study area) in 2100 for the Constant and CAA2025 deposition scenarios.

Using the Return to PE scenario as the point of reference, Fig. 3 shows how N deposition is higher in 2100 across the 19‐state study region for the CAA2025 and Constant deposition scenarios.

### Climate change scenarios

Four climate scenarios, consisting of total annual precipitation and average annual temperature from 2005 to 2100, were applied to the study area USFS FIA plots. Three of these scenarios are based on Representative Concentration Pathways (RCPs), which are the greenhouse gas (GHG) concentration trajectories used by the Intergovernmental Panel on Climate Change (IPCC) in their Annual Report 5 (AR5; IPCC 2014) to represent possible climate futures. The four scenarios are: (1) recent climate, averaged over a 30‐yr period (1981–2010), continued into the future (hereafter referred to as “Constant”), (2) RCP2.6, which is the low‐end GHG concentration trajectory from the IPCC AR5, (3) RCP6.0, which is one of the mid‐range GHG concentration trajectories in the IPCC AR5, and (4) RCP8.5, which is the high‐end GHG concentration trajectory from the IPCC AR5.

Thomas et al. (2010) used PRISM climate data to represent annual mean temperature and annual precipitation by conducting bilinear interpolation of 800‐m resolution climate records (AN81m data set) during the years between FIA plot measurements (data *available online*).^2^ In the interest of using the most comparable climate data set, we selected PRISM 30‐yr average (1981–2010) climate data at 4‐km resolution as the “base” temperature and precipitation data sets for the USFS FIA plots within the study area. The four climate scenarios were then determined by calculating percent change within a scenario and grid cell, which were then multiplied by these “base” climate maps.

**Figure 4:**
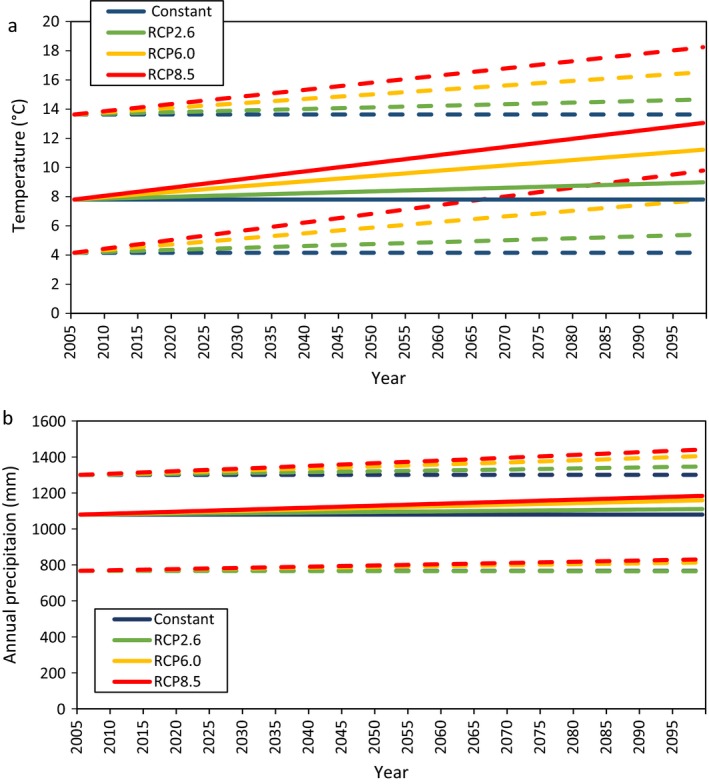
Average annual (a) temperature and (b) precipitation from 2005 to 2100, according to the four climate scenarios. Solid lines represent the 50th percentiles and the dotted lines represent the 5th and 95th percentile of average annual temperature and precipitation across the study area.

**Figure 5:**
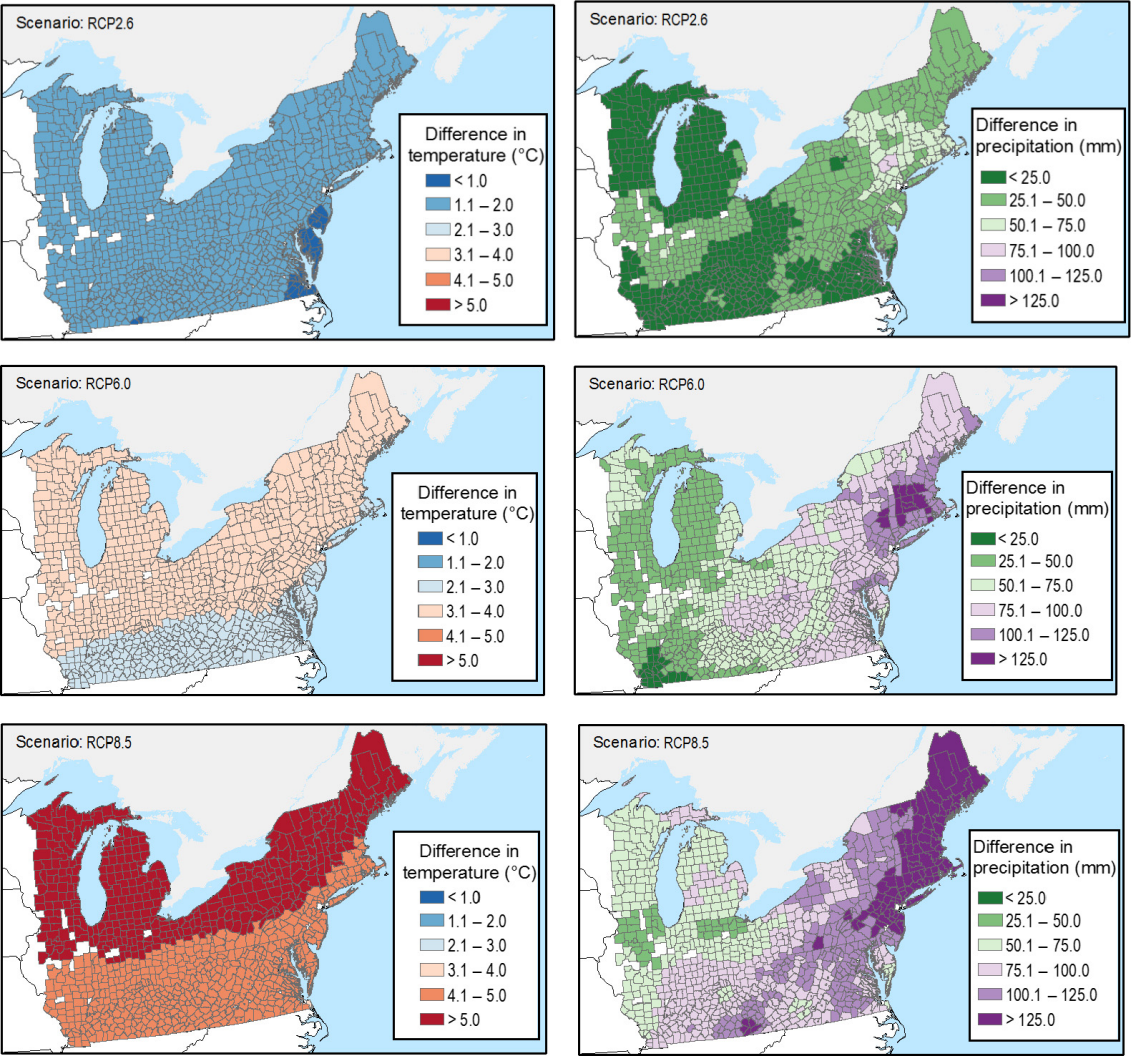
County‐level differences in 2100 for average annual temperature and annual precipitation, relative to the Constant climate scenario.

The Constant climate scenario consisted of repeating the PRISM 30‐yr average annual temperature and precipitation within each grid cell from 2005–2100. The three future IPCC RCP climate scenarios consisted of ~900 m resolution NASA NEX‐DCP30 modeled annual precipitation and temperature translated into percent changes in climate from 2005–2100 (data *available online*).^3^ More specifically, for each of the three RCP scenarios, we applied the plot‐level percent differences between 10‐yr averages (2006–2015 and 2090–2099) of the 50th percentiles of precipitation and temperature estimates from up to 34 climate models to calculate the percent change in precipitation and temperature at each plot. Consistent with the general trends reflected in the NASA NEC RCP climate scenarios, these percent changes were then applied as linear changes in temperature and precipitation from 2006–2100. Thus, we preserved the spatial pattern of precipitation and temperature in the empirical record and the spatial pattern in the relative changes projected in the AR5. Fig. 4a and b present a summary of the temperature and precipitation from 2005–2100 according to the four future climate scenarios. Using the Constant scenario as a reference condition, Fig. 5 shows how temperature and precipitation are projected to increase across the study area for the RCP2.6, RCP6.0, and RCP8.5 scenarios. In contrast to the temperature changes, which are relatively uniform across the study area, the changes in precipitation vary more widely. Across the three scenarios, changes in precipitation are generally larger in counties located farther to the north and east. Maps of baseline (in 2005) average annual temperature and precipitation levels are shown in Appendix S1: Fig. S1.

### Combined N deposition and climate change scenarios

In summary, a total of 12 deposition–climate future scenarios from 2005 to 2100 were evaluated in this study (Table 1). As a point of reference for comparing modeled outcomes at the end of the simulation period (2100), we selected the combination of the Return to pre‐European N deposition and Constant climate (Return to PE/Constant) as the focal “reference scenario.” This deposition–climate combination, which represents a “low pollution” scenario, was chosen as the focal reference scenario to help simplify the interpretation of results. From this reference point, all other scenarios represent larger changes in climate and increases in N deposition.

**Table 1:** Summary of combined N deposition and climate change scenarios

No.	Combined scenario (N deposition/climate change)
1	Return to PE/Constant (reference scenario, with least future N deposition and climate cchange)
2	Return to PE/RCP2.6
3	Return to PE/RCP6.0
4	Return to PE/RCP8.5
5	CAA2025/Constant
6	CAA2025/RCP2.6
7	CAA2025/RCP6.0
8	CAA2025/RCP8.5
9	Constant/Constant
10	Constant/RCP2.6
11	Constant/RCP6.0
12	Constant/RCP8.5 (scenario with most future N deposition and climate change)

*Note*

PE, pre‐European (decline to 0.4 kg N/ha); CAA, Clean Air Act; RCP, Representative Concentration Pathway; Constant refers to remaining at 2005 conditions.

### Forest stand composition model

To model changes in forest stand composition for a single mixed‐age cohort of trees, we applied the species‐specific growth and survival equations, which are described and parameterized in Thomas et al. (2010), to the individual trees in the tree database. These equations related two responses (i.e., annual tree growth and decadal probability of survival over 5 yr) at the individual tree level to four main factors: tree starting size, total annual precipitation, average annual temperature, and average annual N deposition at the plot location. For each scenario, annual changes in biomass were estimated for 24 tree species in the 19‐state study area over the time period from 2005 to 2100. The 24 modeled species represent the most common species across the study area (Thomas et al. 2010). For consistency with Thomas et al. (2010), annual N deposition, temperature, and precipitation estimates from the scenarios were represented as running 10‐yr averages calculated each year, and survival was calculated each year as a 5‐yr probability. The probability estimates were then divided by five to provide annual estimates of survival. Individual tree biomass and number of surviving individuals at the end of one year served as the starting condition of the following year.

Following the calculation of individual tree biomass and survival each year using the Thomas et al. (2010) equations, the values were subsequently expanded to the county level (for each county in the study region) through the FIA expansion factors to provide estimates of forest composition biomass and stem counts from 2005 to 2100. Thus, the forest composition model predicted changes through time in the number of surviving trees of the current northeastern forest cohort (starting in 2003) and their associated biomass. It was not possible to model or include regeneration or ingrowth of new trees and multiple cohorts in this current study, as the Thomas et al. (2010) growth and survival relationships were only developed for trees greater than 12.7 cm at breast height. (1.3 m). Future efforts underway may develop and include models for tree regeneration/recruitment in response to climate and deposition.

In addition to the above model details, several other conditions or factors regarding the forest composition model should be noted. First, to isolate the effect of N deposition and climate change on the composition of a single cohort of trees through time, forest composition projections were made under the assumption that forest area would remain constant over the 95‐yr study periods (i.e., there would be no gain or loss of forested area due to harvesting urbanization, abandonment, or other factors). With continuing population growth in the area and the attendant competing demands for land, this assumption may overstate future forest area, biomass, and ecosystem services. For example, the 2010 USFS Resource Planning Act (RPA) Assessment estimated that 8% of forest land could be lost nationally by 2060 based on the conditions of the IPCC Special Report on Emission Scenarios (SRES) A2 development scenario (USDA Forest Service 2012). Based on where these losses take place, this could have asymmetric effects on individual species because species are not uniformly distributed across the region.

Second, to prevent individual trees from growing beyond observed sizes, all trees were modeled to stop growing (i.e., individual tree growth of a species was set to 0 kg/yr) once they reached the largest recorded biomass for their species within the USFS FIA database. However, only 0.3–0.5% of the trees within our study reached their species‐specific maximum biomass and ceased growth during the 2005–2100 period.

In addition, to prevent our model from extrapolating beyond the empirical records used to develop the Thomas et al. (2010) relationships, we restricted the growth and survival estimates to the observed ranges of the data (within the study area) used to produce the relationships. For all parameters (biomass, N deposition, precipitation, and temperature), once plot conditions were outside the Thomas et al. (2010) empirical ranges for a species, annual growth and survival were modeled to continue at the rate associated with the upper or lower limit for that variable. As the future scenarios progressively deviated from the current deposition and climate conditions, the proportion of trees reaching the upper or lower limits of their empirical ranges of N deposition, temperature, and precipitation increased. The implications of these restrictions are described in *Discussion*.

Third, as described earlier, the Thomas et al. (2010) growth and survival relationships were only developed for trees (i.e., trees with stems >12.7 cm of diameter at a height of 1.3 m), and it was not possible to include the recruitment of new trees and the growth and survival of seedlings in our model. Therefore, only a single cohort of trees was modeled in our study (i.e., only the trees present in the tree database in 2005). We discuss the implications of this in the discussion section.

Lastly, in applying the Thomas et al. (2010) equations, we are transferring the results from a cross‐sectional spatial analysis and applying them to predict changes over time. Although this type of “space‐for‐time” substitution approach is widely used, for example in ecological models of climate change impacts (Blois et al. 2013), it does introduce additional uncertainty to the analysis.

### Ecosystem services

As forest composition changes in response to different N deposition and climate futures, so will the ecosystem services provided. To identify specific ecosystem services provided by each of the 24 species, we began by reviewing the USFS Fire Effects Information System (FEIS), which is a comprehensive resource summarizing the ecology, wildlife uses, and products derived from 1,084 species (*available online*).^4^ We found that over 46 services are associated with the 24 tree species included in this study (see Appendix S1: Table S3), all of which could theoretically either be positively or negatively impacted by altered forest composition. However, it is difficult to quantify changes for many of these services. For example, compositional changes are likely to affect benefits from forest recreation and aesthetics (e.g., fall foliage, bird nesting habitat, etc.), but research and other evidence connecting forest composition to these benefits are limited. Thus, for the purposes of this study, which is a proof‐of‐concept piece, we focused on evaluating and quantifying the impacts on three key services that were more robust to estimate. First, we estimated differences in the amount and value of C that is sequestered in trees from 2005–2100 across the 12 scenarios. Secondly, we examined how the provisioning of merchantable timber is differentially affected across the scenarios. Third, to approximate the many services provided by increased forest biodiversity, we computed tree species diversity across scenarios. In the *Discussion*, we further address and explore the broader set of ecosystem services (Table A.3) that may be impacted by the changes in tree diversity and forest composition.

#### Carbon sequestration

With different future paths of forest growth and compositional change across the 12 scenarios, there will be differences in the amount and value of C that is sequestered through time. This sequestration provides a beneficial service to humanity because it helps to counteract the climate change impacts of C emissions from other sources (USEPA 2005). To compare these benefits across scenarios (*i* = 1–12), we estimated the following “present value” relationship for each scenario. Present value calculations (i.e., discounting) are standard practice in economic analysis for combining monetary cost or benefit estimates from different time periods, which “reflects that people prefer consumption today to future consumption, and that invested capital is productive and provides greater consumption in the future” (USEPA 2010:6‐1)

$${\text{VC}}_{i}=\sum_{t=1}^{19}\left[{\text{SCC}}_{t}*c*\Delta B_{\mathrm{ti}}*{\left(1+r\right)}^{10-\left(t*5\right)}\right]$$

where VC_*i*_ is the present value (US$) in 2015 of total C sequestered from 2005 to 2100 by 24 tree species in the study region under model scenario *i;* *t* is the index for the 19 5‐yr periods from 2005 to 2100; SCC_*t*_ is the social cost of C (US$ per Mg of C) in period *t*;* c* is the C content coefficient (Mg of C per Mg of tree biomass); Δ*B*_*ti*_ is the change in total tree biomass (in Mg) for the 24 tree species in the study area in period *t* and model scenario *i; r* is the annual discount (i.e., interest) rate.

Using our estimates of tree biomass for our modeled cohort of trees under the 12 scenarios, we began by calculating the increase in total biomass Δ*B*_*ti*_ for each 5‐yr period from 2005 to 2100. The 5‐yr increments were selected to simplify the analysis and because the SCC estimates used in the analysis are reported for these periods. Next, to convert these biomass estimates to C and consistent with Thomas et al. (2010), we applied a standard 50% C content of tree biomass (by oven‐dry mass) for all species (Jain et al. 2010).

To estimate the monetary value of sequestered C under each scenario and time period, we applied estimates of the social cost of C (SCC) (United States Government, Interagency Working Group on Social Cost of Carbon, 2016). The SCC is an estimate of the average per‐unit global societal loss associated with each ton of C emitted in a specific year. Because C sequestration can be thought of as a negative C emission; SCC can also be interpreted as an *avoided* cost (i.e., benefit) value per additional unit of C sequestered during the year. The SCC value varies according to the year in which the C is sequestered, because emissions further in the future are expected to be more damaging, as physical and economic systems become more stressed due to future climate change. These per ton values account for the flow of expected future damages from each unit of C emission, by discounting the future damages back to the year of emission. For this analysis, we selected the SCC estimates based on the central assumption of a 3% discount rate (estimates using 2.5% and 5% rates were also presented in the SCC report).

The Interagency Task Force reports SCC values for 5‐yr increments from 2010 to 2050; therefore, we also needed to extrapolate from these values to estimate SCC for the 2055 to 2100 period. Fitting a trend through the SCC estimates for 2010 to 2050 reveals an almost perfectly linear relationship. The linear relationship between SCC (expressed in 2007 U.S. dollars per Mg of CO_2_) and the year (from 2010 to 2050) was estimated to be SCC = 0.96 × year + 1897.1 (*R* ^2^ = 99%). To convert the reported SCC estimates to values per Mg of carbon rather than CO_2_, we multiplied by the ratio of their weights (3.667). To adjust for price inflation, converting from 2007 dollars to 2015 dollars, we multiplied by the ratio of the GDP price index in the 2 yr (1.13). Given this fit, we assumed that the same relationship would continue to hold and we extended through the 2050–2100 period. The resulting SCC estimates vary from US$128.5 per Mg of C sequestered in 2010 to US$480.5 in 2100. However, these values are only discounted back to the year in which the C sequestration occurs. To express them in present value terms in 2015, they were further discounted to 2015 at the same 3% rate. The resulting present values in 2015 vary from US$148.9 per Mg of C sequestered in 2010 to US$39 for C sequestered in 2100.

#### Merchantable timber

Different tree species have different market values as timber. Thus, differences in the tree species composition of forests under alternative deposition and climate change scenario will also have implications for the commercial value of its standing timber resources. This value will differ across species depending on the qualities and attributes of the wood and its suitability for use in different wood products. In *net* terms, it will also depend on the costs of harvesting and extracting the wood for further processing.

Given these differences between species, we posed the following question: will the change in the composition of the standing stock entail a shift toward timber with more or less merchantable value? The long‐time horizon for this analysis (to 2100) made it difficult to predict future demand and supply conditions and resulting prices in timber markets. Instead, we made the simplifying assumption that the *relative* value of the different species, as reflected in their recent market prices, remained constant into the future. Rather than trying to predict future prices and harvest levels, we assessed and compared the value of modeled standing stocks in the future, using currently observed prices. In other words, for each scenario *i* we estimated a “total merchantable value” index (TMV) of the standing stock of the 24 species in 2100 (i.e., treating all forest stock as potentially harvestable at that stage) using the following equation

$${\text{TMV}}_{i}=\sum_{j=1}^{24}P_{j}*V_{\mathrm{ij}}$$

where *P*_*j*_ is the weighted average price (inflation‐adjusted to 2015 dollars) for species *j*;* V*_*i*_ is the volume of species *j* (in thousands of board feet [MBF; 1 board foot = 0.00236 m^3^]) in 2100 under scenario *i*.

In essence, the TMV is a value‐weighted sum of the modeled biomass of the 24 species, where the biomass is converted to a merchantable volume measure.

To estimate TMV, we began by using recent state‐level data to estimate an average price *P*_*j*_ for each species. Most of the states in the 19‐state study region provide summaries of timber prices for selected species; however, there was considerable variation across states in the content and structure of these reports, including the tree species for which prices were reported (Appendix S1: Table S4). Most of these reports included data for recent years (2014–2016). The data were typically reported on an annual or seasonal basis, and they were sometimes reported by sub‐region within the state. We acquired reports for 15 of the 19 states included in the study region. Publicly available species‐specific price data are not available for years more recent than 2010 in Virginia, Maryland, Delaware, or New Jersey. Price data for Connecticut, Massachusetts, and Rhode Island are reported as a single regional group. We summarized these available data by selecting the most recent year of available data on species‐specific stumpage prices (per MBF of saw timber) from each state (or regional group) and estimating average prices for the species of interest. Stumpage prices are the amount paid per unit for the right to harvest standing timber. Depending on how these prices were disaggregated and reported, we averaged across reported sub‐regions and time periods to produce a single price estimate for species with available data in each state (or group of states).

Table 2 provides a summary of collected price data. First, prices for only three species, sugar maple, red maple, and white ash, were reported by all 13 of the states/regions with data. The other most frequently reported species prices were for white oak (12 states), northern red oak (11), and black cherry (10). Second, no prices were reported in any of the states for two species: scarlet oak and sweet birch. Because of this missing price data, we estimated TMV_*i*_ by only summing across the 22 species for which we had data. Due to the relatively low prevalence of the two missing species in the study area and across scenarios (at most 2% for each one), results are very insensitive to whether these species are omitted from the analysis or included using surrogate prices (e.g., prices of other oaks and birches). Third, New York and Wisconsin included data for the largest number of species (17 and 15 species, respectively).

**Table 2:** Average stumpage prices ($/MBF) for selected tree species, ordered from highest to lowest weighted average price

Common name	Genus and species	No. states reporting†	Unweighted average price across states (US$)	State‐biomass‐weighted average price (US$)
Chestnut oak	*Quercus prinus*	2	431	560
Black cherry	*Prunus serotina*	10	441	504
Sugar maple	*Acer saccharum*	13	434	438
White oak	*Quercus alba*	12	354	429
Black oak	*Quercus velutina*	2	344	398
Northern red oak	*Quercus rubra*	10	406	390
Pignut hickory	*Carya glabra*	5	324	311
White ash	*Fraxinus americana*	13	257	259
Yellow poplar	*Liriodendron tulipifera*	8	197	244
Yellow birch	*Betula alleghaniensis*	7	200	209
Red maple	*Acer rubrum*	13	233	188
Red pine	*Pinus resinosa*	6	95	172
American basswood	*Tilia americana*	7	206	163
Red spruce	*Picea rubens*	6	94	122
Eastern white pine	*Pinus strobus*	9	119	118
Balsam fir	*Abies balsamea*	3	104	114
Northern white cedar	*Thuja occidentalis*	3	105	99
Bigtooth aspen	*Populus grandidentata*	2	103	94
Paper birch	*Betula papyrifera*	3	76	68
Eastern hemlock	*Tsuga canadensis*	8	84	62
American beech	*Fagus grandifolia*	7	117	59
Quaking aspen	*Populus tremuloides*	3	53	57
Scarlet oak	*Quercus coccinea*	0		
Sweet birch	*Betula lenta*	0		

*Notes*

See Appendix S1: Table S1 for individual state prices. MBF, thousands of board feet (1 board foot = 0.00236 m^3^).

† Connecticut, Massachusetts, and Rhode Island report together, so are treated as a single state in this table.

Using these state‐level estimates, we next estimated a region‐wide average price for each species (*P*_*j*_). Ideally, we would weight state prices by the volume of timber exchanged in each state to calculate these averages. However, neither price nor timber volume data *for a given species* were consistently available across states. As an alternative approximation of these weights, we used our FIA‐based estimates of the total state‐level biomass for each species in 2005. This weighting by state approximated the effect of more trees of a given species being potentially extracted from a state with a higher (or lower) price driving up (or down) the total market value of merchantable timber for that species. As shown in Table 2, the species with the highest biomass‐weighted average prices per MBF were chestnut oak (US$560), black cherry (US$504), and sugar maple (US$438). The lowest were for quaking aspen (US$57), eastern hemlock (US$62), and paper birch (US$68).

The next step in estimating TMV for our modeled cohort of trees was to estimate *V*_*j*_ by converting the standing biomass estimates for each species (in MT) to merchantable volume (in MBF). This conversion involved three steps. First, we excluded the portion of aboveground biomass that was not merchantable. For this conversion, we used region‐specific ratios of total tree biomass to merchantable tree biomass for softwoods and hardwoods (Birdsey 1992). Second, we converted merchantable weight (Mg) to merchantable timber volume (MBF). For this conversion, we applied the following relationship relating weight to volume (Miles and Smith 2009) for each species:

$$V_{j=}B_{j}/\left(sg_{j}*w\right)$$

where *V*_*j*_ is net volume (cubic feet; 1 cubic foot = 0.028 m^3^) of green wood in the central stem for species *j*;* B*_*j*_ is oven‐dry biomass (pounds; 1 pound = 0.454 kg) of wood for species *j*; sg_*j*_ is “green specific gravity of wood” coefficient for species *j* (Miles and Smith 2009), which is a measure of the wood's density relative to the density of water; *w* is the mass of one cubic foot of water (62.4 pounds).

Finally, we converted metric tons (Mg) of wood biomass to pounds and used the conversion factor of 83.33 cubic feet per MBF to convert wood volume to MBF (U.S. Department of Energy 2011).

It is important to note that C sequestration and the provision of merchantable timber are interconnected ecosystems services that cannot simply be added together. In particular, realizing the merchantable timber benefits requires harvesting trees at some point in the future; however, this process will affect sequestration (depending on how the timber is used and how forests are managed after extraction). As a simplification, our analysis did not account for future harvests and treats the two services as separate.

#### Tree diversity

In many biological communities, changes in species diversity can alter ecosystem properties and functions and the ecosystem services they provide to humans (Hooper et al. 2005, Balvanera et al. 2006). There is evidence of positive relationships between forest tree diversity and several ecosystem service indicators such as soil C storage and tree biomass (Gamfeldt et al. 2013), invasion resistance (Iannone et al. 2016), and nutrient acquisition and cycling (Zeugin et al. 2010), among other processes. We directly estimated tree species diversity for different scenarios and locations using a Shannon diversity (SD) index, recognizing that several intermediate and final ecosystem services may be impacted by changes in tree diversity. SD accounts for both the number and abundance of species in an ecosystem and is calculated as follows:

$${\text{SD}}_{\mathrm{ij}}=\sum_{j=1}^{n_{i}}p_{\mathrm{ij}}*\ln\left(p_{\mathrm{ij}}\right)$$

where *p*_*ij*_ is the proportion of total tree biomass in location *i* (i.e., county or region) that is contributed by tree species *j*;* n*_*i*_ is the number of tree species (from the set of 24 species included in the study) in location *i*.

## Results

### Forest biomass and composition

**Figure 6:**
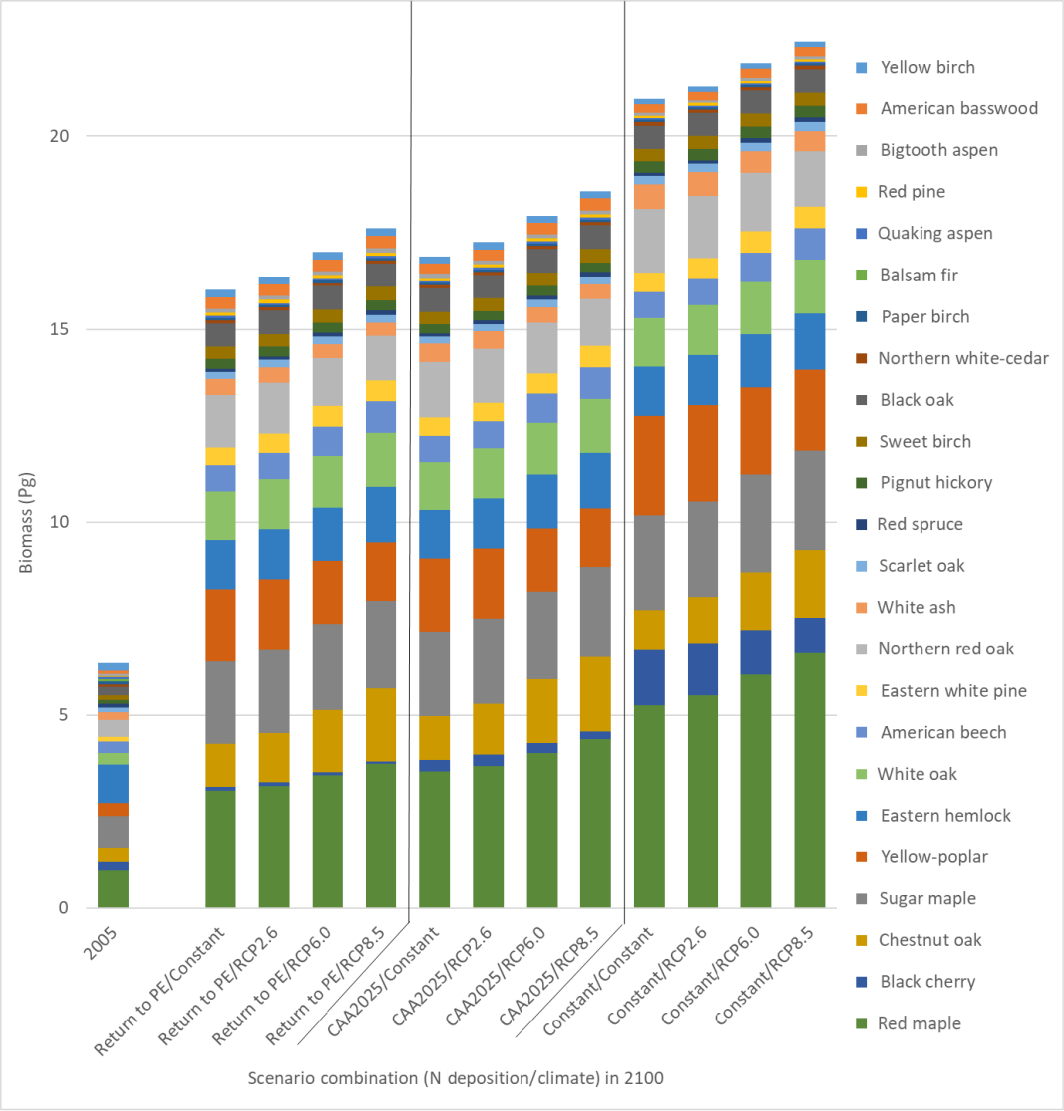
Species‐specific biomass in 2005 and predicted maximum potential biomass of the 2005 cohort in 2100 for the 12 scenarios.

Total aboveground biomass in 2005, the baseline for the modeled cohort and study region, was 6.34 Pg (first bar in Fig. 6). The three dominant species that together accounted for over 44% the total biomass were eastern hemlock (*Tsuga canadensis*; 16%), red maple (*Acer rubrum*; 15%), and sugar maple (*Acer saccharum*; 13%). The five other most prevalent species were northern red oak (*Quercus rubra*; 7%), chestnut oak (*Quercus prinus*; 6%), yellow poplar (*Liriodendron tulipifera*; 5%), white oak (*Quercus alba*; 5%), and American beech (*Fagus grandifolia*; 5%). The remaining 16 species individually represented less than 5% of forest biomass and together accounted for a total of 28%. Overall, broadleaf species were dominant and accounted for 79% of total biomass in 2005.

To analyze how forest composition was influenced by the 12 N deposition–climate scenarios, we estimated total tree biomass and distribution across species in 2100 for each scenario (Fig. 6). The starting basal area (i.e., in 2005) of the FIA plots within the study area ranged from 4.5–40 m^2^/ha (5th–95th percentile) and averaged 22 m^2^/ha, which indicates a relatively young, immature forest condition. Compared to 2005, total aboveground biomass increased by between 9.7 Pg and 16.1 Pg (a factor of between 2.5 and 3.5) across scenarios.

Because the simulations tracked a single mixed‐age cohort of trees over this period, net changes in total biomass from year to year were driven by individual tree growth (positive effect) and mortality (negative effect). Average annual tree growth from 2005 to 2100, which varied between 23–34 kg·tree^−1^·yr^−1^ across scenarios (Table 3), was positively influenced by both N deposition (+4–5 Pg of biomass in 2100 across N deposition scenarios) and climate change (+1–2 Pg of biomass across climate change scenarios). In contrast, total tree mortality, which resulted in the loss of between 60–62% of trees from 2005–2100, was lower under conditions of high N deposition but higher under conditions with more climate change (i.e., larger GHG increases), predominantly due to the projected effects of higher temperatures. Thus, in 2100, there were roughly 130 million fewer trees in the Return to PE scenario compared with the Constant deposition scenario because of lower N deposition, and 200 million fewer trees in RCP8.5 compared with the Constant climate scenario because of higher temperatures.

**Table 3:** Summary of forest biomass and stem count in 2005 and 2100 and percent mortality and average tree growth for the 95‐yr period (2005–2100) by N deposition/climate scenario

Scenario	Biomass (Tg)	Count of stems (millions)	Mortality 2005–2100 (%)	Average annual tree growth 2005–2100 (kg·tree^−1^·yr^−1^)
2005 forest condition	6,341	15,132		
2100 (N Deposition/Climate Scenarios)				
Return to PE/Constant	16,017	5,874	−61.2	23.4
Return to PE /RCP2.6	16,357	5,839	−61.4	24.2
Return to PE/RCP6.0	17,010	5,757	−62.0	25.6
Return to PE/RCP8.5	17,612	5,689	−62.4	26.8
CAA2025/Constant	16,891	5,952	−60.7	24.2
CAA2025/RCP2.6	17,250	5,914	−60.9	25.0
CAA2025/RCP6.0	17,939	5,823	−61.5	26.5
CAA2025/RCP8.5	18,571	5,745	−62.0	27.8
Constant/Constant	20,957	5,996	−60.4	29.6
Constant/RCP2.6	21,296	5,945	−60.7	30.5
Constant/RCP6.0	21,902	5,824	−61.5	32.3
Constant/RCP8.5	22,462	5,718	−62.2	33.8

Overall, the amount of total biomass in 2100, in response to the growth and mortality combined, was positively related to both greater N deposition and climate change, with N deposition being the stronger driver of response. Varying the climate projections from Constant (no change) to RCP8.5 (most change) led to 7–10% more total biomass in 2100 averaged across the three N deposition scenarios (even though stem count declined), whereas higher N deposition projections in the Constant scenario compared to the Return to PE (i.e., largest difference) led to 22–24% more biomass in 2100 averaged across the four climate scenarios.

**Figure 7:**
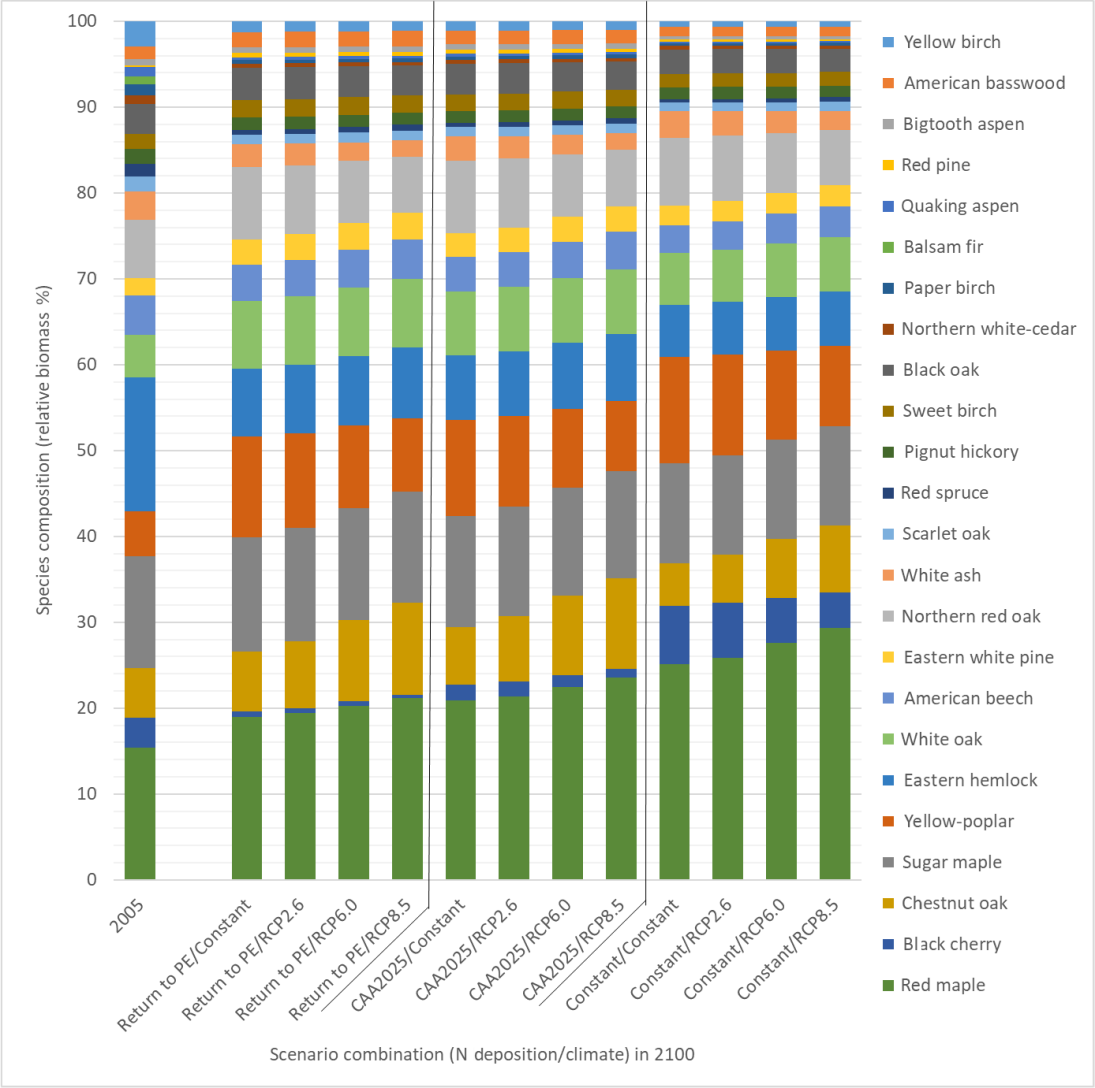
Species composition, as a percentage of total biomass of the 24 tree species, for the 2005 cohort and for the 12 N deposition–climate scenarios in 2100.

Changes in cohort biomass from 2005 to 2100 varied markedly across species (Fig. 6). The species showing the greatest average gains in cohort biomass across all scenarios were red maple (3.4 × 10^12 ^kg), yellow poplar (1.6 × 10^12^ kg), sugar maple (1.5 × 10^12 ^kg), and chestnut oak (1.1 × 10^12 ^kg; Fig. 6). Three species, paper birch (*Betula papyrifera)*, quaking aspen (*Populus tremuloides*), and balsam fir (*Abies balsamea*), lost absolute biomass across all 12 scenarios (average of 1.6 × 10^10 ^kg, 1.9 × 10^10 ^kg, and 6 × 10^10^ kg, respectively). The species showing the greatest average gains in percentage of total biomass (Fig. 7) were red maple (8%) and yellow poplar (5%), and those showing the greatest losses were yellow birch (*Betula alleghaniensis*) and eastern hemlock (loss of 5% and 8%, respectively). Interestingly, eastern hemlock declined the most in relative abundance even though its biomass increased in all scenarios from 2005 to 2100. Its relative decline occurred because of the much larger rates of biomass growth experienced by other tree species. Additional species biomass change comparisons are shown in Appendix S1: Figs. S2 and S3.

Individual tree species responded differently to the N deposition and climate scenarios because of differences in responses and initial conditions (Table 4). Comparing modeled conditions in 2100, the species that showed strongest increases in total biomass when N deposition was higher (i.e., comparing Constant with Return to PE, termed “N‐winners”) included red maple, black cherry, yellow poplar, sugar maple, and northern red oak. In contrast, species whose biomass in 2100 was lower for scenarios with higher N deposition (termed the “N‐losers”) included chestnut oak, American basswood (*Tilia americana*), yellow birch, bigtooth aspen (*Populus grandidentata*), and red pine (*Pinus resinosa*). Several other species showed relatively little to no responses to N deposition, including, white oak, American beech, black oak (*Quercus velutina*), sweet birch (*Betula lenta*), and red spruce (*Picea rubens*). Note that these shorthand names refer to how the species responds to an increase in the stressor, not how it responds to expected future changes in the stressor (which, as in the case of N deposition, could be negative).

**Table 4:** Tree species most responsive to N deposition and/or climate change

Species	Difference in biomass (kg)		
	N deposition sensitivity, Return‐to‐PE to constant†	Climate sensitivity, Constant to RCP8.5‡	Combined sensitivity, PE/constant to constant/RCP8.5
Red maple	*2.50* × *10* ^*12*^	*9.56 × 10* ^*11*^	*3.55 × 10* ^*12*^
Black cherry	*1.13 × 10* ^*12*^	**−2.18 × 10** ^**11**^	*8.18 × 10* ^*11*^
Chestnut oak	**−9.93 × 10** ^**10**^	*7.65 × 10* ^*11*^	*6.44 × 10* ^*11*^
Sugar maple	*3.08 × 10* ^*11*^	*1.49 × 10* ^*11*^	*4.62 × 10* ^*11*^
Yellow poplar	*6.59 × 10* ^*11*^	**−4.17 × 10** ^**11**^	*2.12 × 10* ^*11*^
Eastern hemlock	0	*1.74 × 10* ^*11*^	*1.74 × 10* ^*11*^
White oak	0	*1.46 × 10* ^*11*^	*1.46 × 10* ^*11*^
American beech	0	*1.35 × 10* ^*11*^	*1.35 × 10* ^*11*^
Eastern white pine	*1.74 × 10* ^*10*^	*8.27 × 10* ^*10*^	*1.01 × 10* ^*11*^
Northern red oak	*2.96 × 10* ^*11*^	**−2.14 × 10** ^**11**^	*7.78 × 10* ^*10*^
White ash	*1.92 × 10* ^*11*^	**−1.15 × 10** ^**11**^	*7.13 × 10* ^*10*^
Scarlet oak	*3.43 × 10* ^*10*^	*2.41 × 10* ^*10*^	*5.92 × 10* ^*10*^
Pignut hickory	*4.93 × 10* ^*10*^	0	*4.93 × 10* ^*10*^
Red spruce	7.97 × 10^8^	*4.63 × 10* ^*10*^	*4.71 × 10* ^*10*^
Sweet birch	0	*3.57 × 10* ^*10*^	*3.57 × 10* ^*10*^
Black oak	0	*9.12 × 10* ^*9*^	*8.50 × 10* ^*9*^
Northern white cedar	*1.34 × 10* ^*10*^	**−5.82 × 10** ^**9**^	*7.58 × 10* ^*9*^
Paper birch	**−5.39 × 10** ^**9**^	*8.50 × 10* ^*9*^	*3.56 × 10* ^*9*^
Balsam fir	4.42 × 10^8^	9.95 × 10^6^	4.54 × 10^8^
Quaking aspen	**−1.50 × 10** ^**10**^	*2.56 × 10* ^*9*^	**−1.25 × 10** ^**10**^
Red pine	**−1.87 × 10** ^**10**^	**−**3.62 × 10^9^	**−2.22 × 10** ^**10**^
Bigtooth aspen	**−3.60 × 10** ^**10**^	*2.13 × 10* ^*9*^	**−3.40 × 10** ^**10**^
American basswood	**−6.56 × 10** ^**10**^	*3.21 × 10* ^*10*^	**−3.43 × 10** ^**10**^
Yellow birch	**−5.74 × 10** ^**10**^	**−**9.13 × 10^8^	**−5.83 × 10** ^**10**^

*Notes*

Values >1 billion kg (10^9^) are shown in italic font and values <−1 billion kg are shown in boldface font ordered (from highest to lowest) according to combined sensitivity.

† Averaged across four climate change scenarios in 2100.

‡ Averaged across three N deposition scenarios in 2100.

With regard to climate scenarios, the species showing the largest positive differences in biomass in 2100 when going from the Constant climate scenario to the RCP8.5 scenario (termed “climate winners”) included red maple and chestnut oak, whereas the species that had less total biomass in 2100 under scenarios with more climate change (termed “climate losers”) included yellow poplar, northern red oak, black cherry, red pine, white ash (*Fraxinus Americana*), northern white cedar (*Thuja occidentalis*), and yellow birch (Table 4). Thus, red maple increased with more of both stressors, yellow birch and red pine decreased with more of both stressors, and chestnut oak had countervailing responses (i.e., N‐winners, climate losers). Total biomass for two species, pignut hickory (*Carya glabra*) and balsam fir, was relatively unresponsive to differences in climate.

Differences were also observed across species in their sensitivity to the combined effects of N deposition and climate change. Going from the combined scenario with the lowest N deposition and least climate change (Return to PE/Constant; Table 4) to the one with highest deposition and most climate change (Constant/RCP8.5), five species end up with lower biomass: American basswood, bigtooth aspen, quaking aspen, red pine and yellow birch. All species were projected to decrease by 10 Tg or more. Red maple was projected to have the largest increase in biomass, increasing by over 3 Pg. Differences in the relative abundance of each species in each county, comparing RCP 8.5/Constant scenario (highest climate change and N deposition) and the Constant/Return to PE scenario (lowest climate and N deposition) in 2100 are shown in Appendix S1: Fig. S4.

### Ecosystem services

#### Carbon sequestration

**Figure 8:**
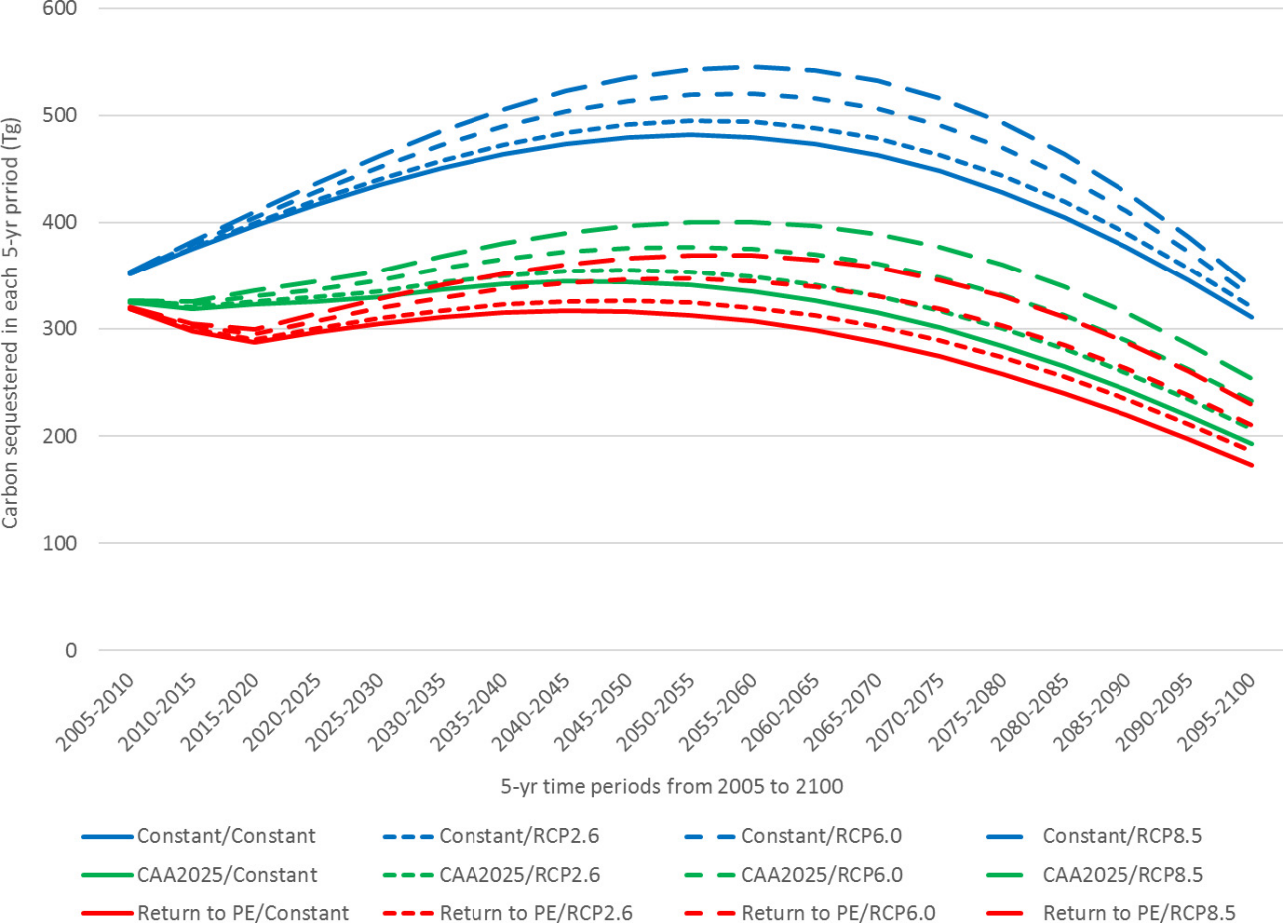
Carbon sequestration (in Tg per 5 yr) in each 5‐yr period from 2005 to 2100.

For each of the 12 future N deposition–climate scenarios, the trajectory of C sequestration in 5‐yr increments was estimated for the modeled cohort (Fig. 8). The highest levels of sequestration occurred in the scenarios that included the Constant (high) N deposition scenarios, and more climate change (higher temp and precipitation; RCP 8.5). The scenarios with lower N deposition, and smaller changes in temperature and precipitation, resulted in less sequestration.

Although sequestration occurred in each scenario and each period, the rate of C accumulation actually declined from 2005 to 2025 for the scenarios involving a reduction in N deposition (CAA2025 and Return to PE). This decline occurred because N deposition fell in these scenarios over the first 20 yr (Fig. 2), before flattening out for the remainder of the simulation period. The flat period of deposition was still associated with increasing C stocks as forests continued to mature. In all scenarios, the 5‐yr rate of C sequestration peaked in the mid‐range of the simulation period (2040–2055) before the cumulative effects of cohort mortality had begun to accumulate.

Compared to the Return to PE/Constant, in which 5.3 Zg were sequestered from 2005 to 2100, total C sequestration was 67% greater in the scenario with the highest levels of N deposition and the greatest climate change (Constant/RCP8.5; Table 5). Consistent with the trends in biomass accumulation, differences in N deposition across scenarios had a larger effect on C accumulation in 2100 than differences in climate change. The Constant N deposition scenarios increased sequestration by 51% relative to the Return to PE scenarios, whereas increasing climate change alone to RCP8.5 increased sequestration by 16%.

**Table 5:** Total C sequestration from 2005 to 2100: percent difference relative to the reference scenario (Return to PE/Constant)

Nitrogen deposition scenario	Climate scenario			
	Constant	RCP2.6	RCP6.0	RCP8.5
Return to PE	0%	4%	10%	16%
CAA2025	9%	13%	20%	26%
Constant	51%	55%	61%	67%

**Figure 9:**
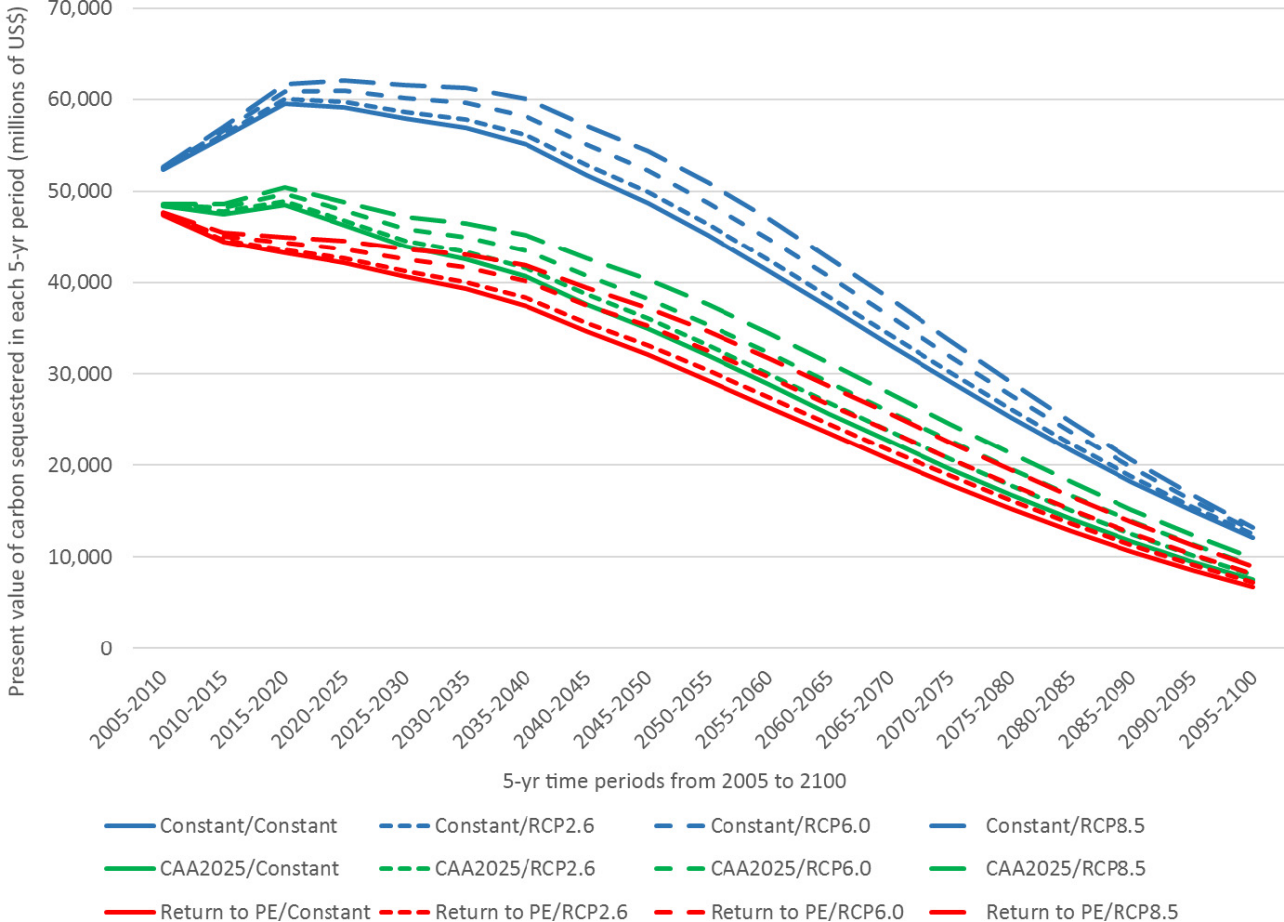
Present value (in 2015) of C sequestration in each 5‐yr period (in millions of US$ per 5 yr).

The present value (in US$) of C sequestration was estimated for each scenario in each 5‐yr period after applying period‐specific SCC values (in 5‐yr increments) to each unit of C sequestered. Because benefits accruing farther in the future have less value from a present day perspective, we discounted all future benefits from sequestration back to the reference year 2015 using a 3% discount rate (Fig. 9). Therefore, the present value estimates represent the time‐discounted sum of avoided future climate change damages by removing and storing C from the atmosphere. For all scenarios, these present values reached a peak several years earlier than the peaks in C sequestration (in Fig. 8) due to the effects of discounting, which can have a larger negative effect on present values than the positive effect of increasing C sequestration.

Comparing the present values of C sequestration across scenarios for the modeled cohort of trees (Table 6), we found that, compared to the Return to PE/Constant scenario with a present value of US$533 billion, the present value of C sequestration was 58% higher in the scenario with most N deposition and climate change (Constant/RCP8.5). Once again, differences in N deposition across scenarios had a larger effect on the present value of C accumulation than differences in climate change. Increasing N deposition to Constant levels relative to the Return to PE levels increased present value by 46%, whereas increasing temperatures and precipitation to RCP8.5 conditions increased it by 13%.

**Table 6:** Present economic value of C sequestration from 2005 to 2100: percent difference relative to the reference scenario (Return to PE/Constant)

Nitrogen deposition scenario	Climate scenario			
	Constant	RCP2.6	RCP6.0	RCP8.5
Return to PE	0%	3%	8%	13%
CAA2025	9%	11%	17%	22%
Constant	46%	48%	54%	58%

#### Merchantable timber

**Figure 10:**
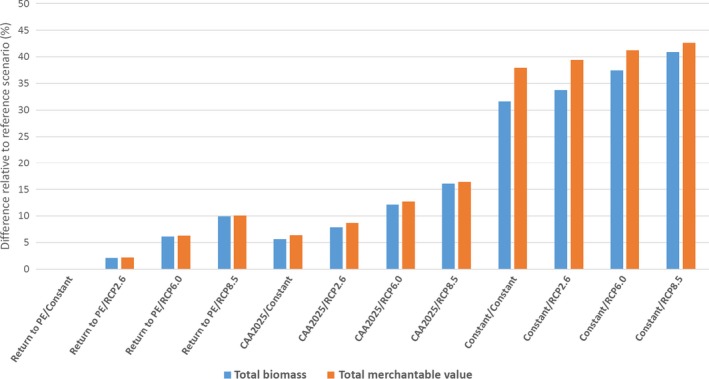
Percent difference in total merchantable value (TMV) and total biomass relative to the reference (Return to PE/Constant) scenario in 2100.

The provisioning services provided by forests through their stock of merchantable timber also varied across N deposition and climate change scenarios, depending in part on how the scenarios shifted forest composition toward or away from species with more or less commercial value. To capture these differences, we estimated how both the total tree biomass (in Mg) and the TMV index for each scenario compared to the reference scenario (Return to PE/Constant) in 2100 (Fig. 10). Because of missing price data for 2 of the 24 species, scarlet oak and sweet birch, which account for 2.5% of total biomass in 2005, these estimates for biomass and TMV only included the 22 species with available price data. As for total biomass, we found that TMV was positively affected by N deposition and climate change; however, the *magnitudes* of the deviations from the reference scenario were somewhat different. In the scenarios with the highest N deposition (Constant), the positive deviations from the reference scenario were always predicted to be greater for TMV (orange bars) than for total biomass (blue bars), ranging from 1 to 5 percentage points higher. These results indicate that the species with the largest increases in biomass with respect to increased N deposition levels also tended to be the higher priced species., such as black cherry, sugar maple, yellow poplar, white ash, and northern red oak. The opposite effect was observed for the scenarios with more climate change. When only climate changes from the reference scenario, TMV increased at a lower rate than total biomass. For example, going from the Constant/Constant scenario to the Constant/RCP8.5 scenario, total biomass increased by 9 percentage points, compared to 5 percentage points for TMV. In this case, several higher priced species, such as black cherry and yellow poplar, were negatively affected by scenarios with the most climate change, and lower priced species such as eastern hemlock and American beech were among the most positively affected.

#### Tree diversity

**Figure 11:**
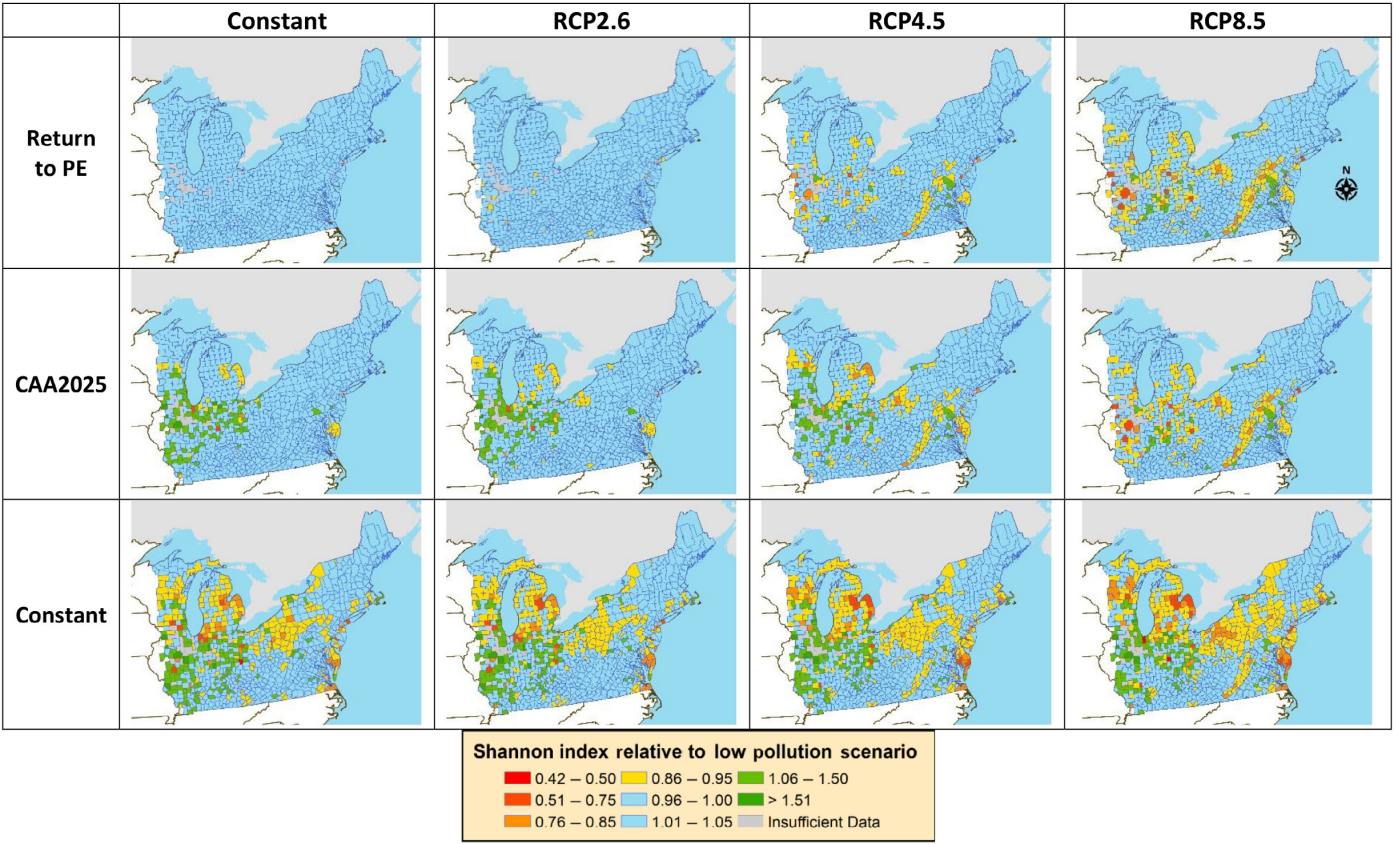
Variation in county‐level tree species diversity relative to reference scenario (Return to PE/Constant).

By examining the ratio of the Shannon diversity index for each county and scenario relative to the Return to PE/Constant scenario (Fig. 11), we found that tree diversity responses varied regionally and across scenarios. The higher RCP scenarios resulted in lower predicted tree diversity in several counties of New York, Pennsylvania, Michigan, Wisconsin, Illinois, and along the Appalachian spine of Virginia, and increased tree diversity in a few small pockets of mostly Indiana and Maryland. Elevated N deposition was predicted to increase tree diversity in Illinois and Indiana and decrease diversity in the same states negatively impacted by climate. As with the other responses, N deposition appeared to have a larger magnitude of effect compared with climate change, leading to an overall increase in diversity in Illinois and Indiana when both stressors were high (Constant/RCP8.5), and an additive decrease in diversity for other regions. Generally, tree diversity in the New England region was unresponsive to either stressor because of offsetting responses at the species level (Appendix S1: Fig. S4).

**Figure 12:**
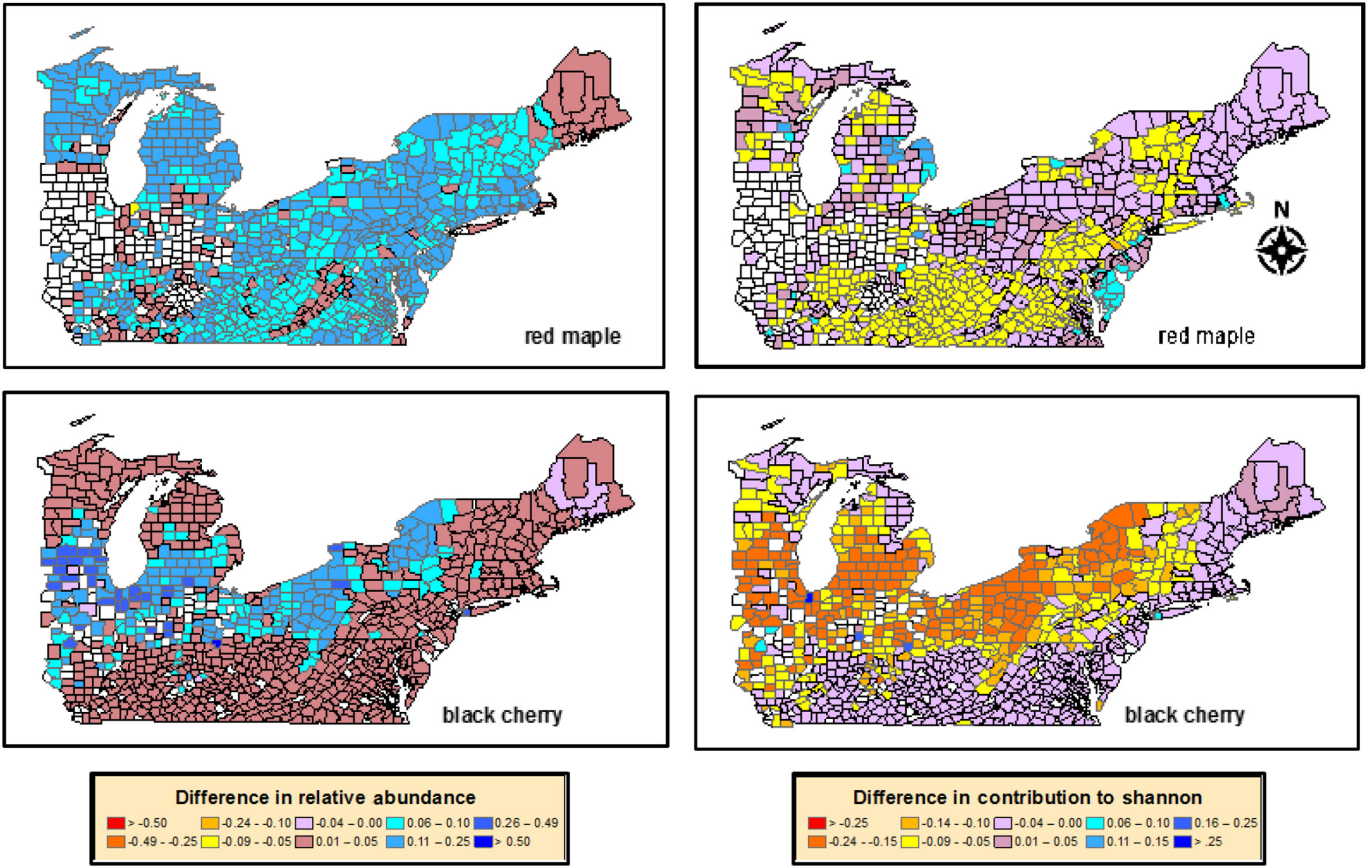
County‐level change in relative abundance (left column) and contribution to changes in Shannon index (right column) for red maple (top row) and black cherry (bottom row). Changes are between the high pollution scenario (RCP 8.5/Constant) and the reference scenario (Constant/Return to PE).

Regional differences in tree diversity were due to differential responses of forest communities across the landscape. For regions that decreased in diversity, this was mostly from increased dominance of black cherry due to higher N deposition, and to a lesser degree from red maple (Fig. 12). These were the two strongest N‐responders and combined responders (Table 4), and their increases came at the expense of several other species. Red maple was already relatively abundant, so further increases in its abundance decreased diversity by reducing species evenness. Black cherry, on the other hand, was rarer under the Return to PE/Constant scenario but was predicted to increase with higher deposition, which would competitively exclude other species. For regions with predicted increased tree diversity, this effect was largely due to decreases in American basswood, black oak, and red oak. These species are relatively abundant in the midwestern portion of the study area; thus, their decrease would increase Shannon diversity due to a decrease in dominance by these species. It should also be noted that red maple is largely absent in this midwestern region under both current and future projected conditions, and the absence of this otherwise dominant species also likely contributed the large changes in diversity in that region of the study area. Other species also contributed to these predicted changes in diversity, but their contributions were more localized or scattered (Appendix S1: Fig. S4).

## Discussion

### Effects on forest biomass and composition

The findings of this analysis regarding forest biomass and composition have both similarities and differences compared to other analyses evaluating the impacts of N deposition and climate change, but direct comparisons are difficult due to differences in objectives, models, and the drivers included. For example, Aber and Driscoll (1997) evaluated the impacts of climate, N deposition, and land‐use and disturbance history on N dynamics and total C storage by forest ecosystems. They found that increasing background N deposition to current levels increased net ecosystem productivity in all the forests by 24–47 g C·m^−2^·yr^−1^; similar differences in net productivity were also found in their comparison of average (1980–1994) vs. real climate conditions. Townsend et al. (1996), modeled the influence of anthropogenic N deposition on global C accumulation from 1845 to 1990, and estimated that in 1990 C storage was 0.44–0.74 Pg C/yr with the rate varying due to different in N deposition level, vegetation type and land‐use. Other studies have focused specifically on forests and the influences of climate change on biomass and composition. For example, Thompson et al. (2011) modeled changes in Massachusetts forest biomass and composition in response to changes in land use and climate. Similar to our study, they found that changes in climate increased total biomass, but they found little impact of climate and land use changes on overall forest composition. Scheller and Mladenoff (2005) also found that Wisconsin forest biomass would increase in response to future climate scenarios. Live biomass was predicted to be 5–29% higher by 2190 if the influence of other disturbances (wind and harvesting) were not accounted for. However, their study also predicted changes in forest composition and varying effects on biomass due to the combined effects of tree species extirpation (poor seed dispersal and migration), harvesting, windthrow, and climate change. In contrast, Duveneck et al. (2014) in their evaluation of the impacts of future climate scenarios, harvest, wind, and fire on northern Great Lakes forests reported lower total forest biomass under the climate associated with the highest emissions scenario. However, they also found that forest productivity and diversity responses and relationships varied with soils, landscape, and regional climate under a high emissions scenario.

The modeled increase in biomass by a factor of between 2.5 and 3.5 from 2005 to 2100 is reasonable for an aggrading forest such as the northeastern United States. For instance, reported basal areas for old growth and mature stands in the northeastern United States range from 16 to 147 m^2^/ha (Leak et al. 1987, Keddy and Place 1994, Tyrrell et al. 1998, Ducey et al. 2013), which is roughly 3.5 times larger than the starting basal area (i.e., in 2005) of the FIA plots within the study area.

### Effects on forest ecosystem services

The wide range of forest composition shifts estimated in this study under alternative N deposition and climate change scenarios has varying implications for forest ecosystem services. Some ecosystem services, such as C sequestration, are positively associated with total biomass accumulation, which was positively associated with both N deposition and climate change in our cohort simulations. This is not surprising since N is often a limiting nutrient in many terrestrial ecosystems. Furthermore, climate change in the northeast is expected to make conditions warmer and wetter, which is often observed with increases in productivity (Nemani et al. 2003, Kirilenko and Sedjo 2007, Chertov et al. 2010). This does not preclude other negative effects from climate factors not accounted for in this study (e.g., increased drought, fire, pests, etc.), and should only be interpreted as the projected impacts from changes in mean climate conditions.

The finding that the rate of biomass accumulation and C sequestration would continue to grow over the first half of the study period is consistent with the observed patterns reforestation and accrual of biomass in the northeastern United States following agricultural abandonment in the 1800–1900s (Williams 1989, Ramankutty et al. 2010).

Other services, such as merchantable timber were predicted to have more mixed effects. Whereas total timber values increased with overall biomass, the scenarios with more climate change tended to favor species with lower merchantable value. This result is broadly consistent with estimates for European forests (Hanewinkel et al. 2013), which indicated a shift toward forest types with lower economic returns as climate continues to change over the next century. The opposite effect was observed for the scenarios with more N deposition; this suggests that we will be losing an unanticipated benefit of poor air quality in the future.

A wide variety of tree diversity responses were also predicted across the region, with increases in some central midwestern states and decreases in northern midwestern states and eastern states (Fig. 11). The implications of this variation depend on the ecosystem function or service of interest. For ecosystem services that are positively associated with tree diversity (e.g., soil carbon storage; Gamfeldt et al. 2013), lower diversity is expected to entail less services. For ecosystem services that are more associated with an individual species (e.g., maple syrup from sugar maple), then less diversification that favors this individual species can increase services. Teasing apart these complexities for a wider range of services and species is an area of future work.

Because our analysis focused on only three main types of ecosystem services that can be readily quantified for our study area, it is important to note that individually affected trees species may provide a variety of other services, many of which may be differently affected by N deposition or climate change (Table A3). However, these changes in services are much more difficult to quantify. For example, three species experiencing the greatest negative effects from higher levels of N deposition (measured as a percentage decline in their biomass) were quaking aspen, yellow birch, and bigtooth aspen. Quaking aspen trees are widely appreciated for their aesthetic qualities, including their light bark and bright fall foliage (Perala 1990, Howard 1996). Materials from the tree have also had several traditional uses by Native American populations including as medicine, food, building material, and fiber for clothing. (The Native American Ethnobotany Database [NAEDB]; *available online*).^5^ Yellow birch is also known for its bright yellow foliage in the fall, which provides aesthetic services to viewers and recreationists (Erdmann 1990, Sullivan 1994). Bigtooth aspen are used, appreciated, and valued by humans in a variety of ways including through recreational activities and existence values (Laidly 1990, Carey 1994). Two of the tree species projected to experience the greatest negative effects from higher levels of climate change were black cherry and white ash (Table 4). In addition to providing wood that is highly valued for cabinet and furniture making, a number of traditional uses have been documented for black cherry, including providing an edible fruit for wines and jellies (Marquis 1990). White ash is particularly well known for its use in baseball bats (Irvine et al. 2017). Because the above ecosystem services were not examined in our study, the overall effect on ecosystem services remains unknown until a complete accounting can be performed. Nonetheless, this study demonstrates how the effects of N deposition and climate change on forests can be species specific and that this variation is predicted to change at least a subset of key ecosystem services provided by northeastern forests.

### Policy implications

These findings underscore the tradeoffs that must be considered when evaluating policies to manage the effects of N deposition and climate change on forest ecosystems and the services they provide. Overall, policies to limit N deposition and climate change are expected to limit growth in total biomass for the current mixed‐age cohort of forest trees in the northeastern United States and the directly associated ecosystem services such as C sequestration. However, this trend may not be ubiquitous across the United States, as forests in other areas may show more consistently negative responses to changes in climate and high levels of N deposition. The reduction in tree diversity associated with greater changes in climate could reduce many ecosystem services not explicitly assessed, and especially those associated with species projected to decrease with climate change (especially yellow poplar, black cherry, and white ash). Furthermore, in the northeast, the effects of forest composition change on ecosystem services are predicted to be very mixed. This is because individual species can provide different combinations of services and because compositional changes can both increase and decrease diversity. In some instances, humans may even associate negative value with any alteration in the tree species composition with which they are familiar (Meyerhoff and Liebe 2009).

### Uncertainties and caveats

In interpreting the results from this study, it important to recognize certain inherent constraints and uncertainties. First, in examining the effects of climate change on forests, this study only addressed the effects of projected changes in mean annual temperature and mean annual precipitation. It did not include many other potential factors, including episodic shifts in temperature and precipitation (e.g., drought, seasonal changes in precipitation timing or amount), potential CO_2_ fertilization effects on tree growth, or other effects that may be influenced by climate or correlated with other factors (e.g., fire, ozone damage, sulfur deposition, pests), which may prove to have significant long‐term effects. For example, some pathogens, such as wooly adelgid (*Adelges tsugae*), can be particularly devastating to certain tree species (Orwig et al. 2002). Understanding how forests in the future will provide ecosystem services requires an understanding of each of these potential factors individually and in combination.

Second, our approach for projecting forest growth and change in associated ecosystem services into the future is a simplified representation of these processes. It uses only the mean response estimates from Thomas et al. (2010), without accounting for parameter uncertainty or uncertainties from applying estimates based on spatial variation to assess changes over time. Also, by modeling the growth and survival of a single mixed‐age cohort of trees, the results only reflect the responses of this single population of trees and not the forest in its entirety. Our analysis did not factor in the influences of climate and N deposition on the ingrowth of new trees (or new tree species due to range shifts) and how ecosystem services might be further impacted by changes in sapling recruitment. Furthermore, other factors such as disturbance, and light gaps known to affect forest growth and dynamics were not accounted for in our model predictions. A process‐based dynamic forest model (e.g., ED2; Medvigy et al. 2012) could be used to add complexity to these estimates. Many of these models, however, aggregate species into broad functional groups (e.g. broadleaf vs. coniferous), and thus cannot be used directly at present to look at species‐level changes in forests. However, comparing our species‐level responses aggregated to common functional groups could yield a basis for comparison with forest dynamic models. In addition, our analysis does not account for future forest management activities that which may contribute to forest turnover and limit biomass growth and carbon sequestration.

Third, the restrictions imposed by our model on the growth and survival estimates of tree species to their observed ranges imply that the estimated biomass responses are conservative. For all parameters (biomass, N deposition, precipitation, and temperature), once plot conditions were outside the Thomas et al. (2010) empirical ranges for a species, annual growth and survival were modeled to continue at the rate associated with the upper or lower limit for that variable. As the future scenarios progressively deviated from the current deposition and climate conditions, the proportion of trees reaching the upper or lower limits of their empirical ranges of N deposition, temperature and precipitation increased. For example, by 2025 (the last year with N deposition changing), 53% of all trees exposed to CAA2025 deposition were experiencing N deposition lower than lowest N deposition reported by Thomas et al. (2010). For individual species, the percent with lower deposition ranged from 7% (black cherry [*Prunus serotina*]) to 100% (scarlet oak [*Quercus coccinea*]) (Appendix S1: Table S1). In response to the Return to PE scenario, despite differences across species in their timing of reaching lower limits, 100% of all trees and species had hit the lowest N deposition level by 2025. Therefore, the effective lowest N deposition level in this scenario was above 0.4 kg N/ha for all species (ranging from 3.4 kg N/ha to 5.4 kg N/ha across species).

Similar conditions were seen in response to the three RCP climate futures. For temperature, by 2100 the percent of all trees experiencing conditions outside of the Thomas et al. (2010) empirical ranges varied from 2.6% for RCP2.6 to 39% for RCP8.5. Across the individual species and scenarios, the percent experiencing temperatures lower or higher than those used to model the species responses varied from 0% to 100% (Appendix S1: Table S2). Predicted changes in future total annual precipitation were small by comparison, and only 2–4% of all trees were exposed to amounts of precipitation outside the empirical ranges of Thomas et al. (2010) (Appendix S1: Table S2). Refer to the referenced tables in Appendix S1 for specific information regarding the N deposition and climate conditions experienced by each species.

Consequently, the actual responses to lower N levels and higher temperatures would most likely result in even larger growth and survival responses relative to current conditions and thus larger potential changes in species composition. However, it is not possible to determine the magnitude of these biomass responses or their potential impacts on ecosystem services. We considered extrapolating the empirical relationships from Thomas et al. (2010) beyond their original range but decided that this introduced more uncertainty than fixing the responses at the edges of the empirical range.

Fourth, in examining and comparing benefits from C sequestration, the analysis did not account for changes in belowground C, which may also be affected by N availability and climate change. Nor did the analysis account for any feedback effects that sequestration may have on the climate change scenarios. In other words, the analysis estimated how alternative climate change predictions could affect rates of C sequestration, but it did not adjust the climate prediction to account of the effects of sequestration. Nevertheless, this feedback effect is not expected to be large because in the model simulations C sequestration was at most about 100 Tg/yr, which is only about 2% of current annual U.S. C emissions (USEPA 2018a *,* b). Indeed, the entire land use, land‐use change, and forestry (LULUCF) sector sink is roughly 11% of the total U.S. GHG emissions; thus, the contribution from the 19‐state area in the Northeast that we examined is expected to be a fraction of that.

Finally, to monetize these ecosystems services we applied simple unit value estimates, SCC values for C and stumpage prices for timber, which each have large ranges of uncertainty, particularly when projected farther into the future. For example, using recently observed timber prices (averaged over states in the study area) to assess the relative values of merchantable timber in the future assumes that the relative demand for and costs of harvesting the species will be similar in the future. As global forest stocks, human tastes, and harvest technologies evolve in the future, these relative prices are likely to change, but in ways that are difficult to predict.

## Conclusion

The primary objective of this study was to develop a proof of concept analysis to assess the effect of N deposition and climate change on forest composition and ecosystems services, with a case study focus on the northeastern United States. As expected, due to the wide variation in how individual species respond to changes in N deposition and climate, large differences in forest composition were predicted across scenarios by the end of the study period (2100). For example, the relative and absolute abundance of red maple trees were notably higher under both high N deposition and high climate change scenarios, whereas yellow birch and red pine were negatively affected in both cases. The effects on forest ecosystem service also varied significantly across scenarios. Carbon sequestration, which is positively associated with total biomass accumulation, was higher in scenarios involving more N deposition and increased climate change. For merchantable timber, whereas total timber values increased with overall biomass, the scenarios with more climate change tended to favor species with lower merchantable value, and the scenarios with more N deposition tended to favor species with higher merchantable value. With regard to tree species diversity, climate change was found to have a mostly negative influence on adult tree species diversity, especially in the northwestern, central, and southeastern portions of the study area. The effects of N deposition on diversity were mixed across the study area, increasing diversity in parts of the Midwest, and adding to the negative effect of climate in parts of the east and northern Midwest. The variable effects of N deposition on tree diversity are more difficult to manage and suggest a species‐level approach may be more advantageous.

A second objective of this study was to identify research areas for extending and improving this approach. One research priority is to expand the model to include more regions, tree species, and environmental stressors such as sulfur deposition and ozone. Another is to improve the tree composition estimates to include the influences of climate and atmospheric deposition on recruitment and ingrowth of new trees, to enable a multi‐cohort study. Another research priority should be to explore and ideally quantify how changes in forest composition could affect other ecosystem services. These other services could, for example, include aesthetic services such as those derived from fall foliage, regulating services such as erosion control and water storage in forested areas, or improved resistance to disease and pests.

Overall, the information provided in this study can inform decision makers on how northeastern forests may respond to dual stressors of N deposition and climate change over this century, and potentially be used to manage forests and their attendant ecosystem services in the face of these changes. Ecosystem services have been specifically mentioned in the EPA's review of the secondary (welfare‐based) standards for oxides of nitrogen, oxides of sulfur, and particulate matter (USEPA 2018a *,* b. This study contributes to that review process and provides a blueprint for future analyses on how to estimate effects from changes in air quality on issues of public concern. We also believe this study can be a model for future efforts aimed at improving predictions of how forests may respond to shifts in deposition and climate. These types of efforts will be essential for informing environmental policies directly affecting the health and productivity of forest ecosystems.

## Data Availability

Data are available from the EPA's Environmental Dataset Gateway: forest composition data, https://doi.org/10.23719/1500027; ecosystem services data, https://doi.org/10.23719/1503110

## Notes

1
https://www.epa.gov/cmaq/cmaq-data#CMAQ-data-files.

2
http://www.prism.oregonstate.edu/

3
https://cds.nccs.nasa.gov/wp-content/uploads/2014/04/NEX-DCP30_Tech_Note_v0.pdf

4
https://www.feis-crs.org/feis/

5
http://naeb.brit.org/
