# Supplementary material for: Nitrogen deposition and climate change effects on tree species composition and ecosystem services for a forest cohort
Source: Ecol Monogr. 2019 Feb 5;89(2):e01345. doi: 10.1002/ecm.1345 (PMC6559268; doi:10.1002/ecm.1345)
Supplement: Supplementary file 1 [file ECM-89-na-s001.pdf]

Supporting Information.

Van Houtven, G., J. Phelan, C. Clark, R. Sabo, J. Buckley, R. Q. Thomas, K. Horn, and S. D. LeDuc. Nitrogen deposition and climate change effects on tree species composition and ecosystem services for a forest cohort. Ecological Monographs. 2018.

Appendix S1

As described and referenced in the main text, this appendix contains tables and figures with additional details regarding the study's methods and results.

Table S1. Proportion of stems, by species and scenario, that experienced nitrogen deposition levels greater than or less than the range of deposition used by Thomas et al. (2010) to develop the species-specific growth and survival relationships.

Species	N Deposition Scenario		
	**Constant**	**CAA2025**	**Return to PE**
American basswood	1%	23%	100%
American beech	0%	33%	100%
Balsam fir	8%	40%	100%
Bigtooth aspen	1%	15%	100%
Black cherry	0%	7%	100%
Black oak	0%	35%	100%
Chestnut oak	0%	46%	100%
Eastern hemlock	14%	76%	100%
Eastern white pine	25%	97%	100%
Northern red oak	0%	43%	100%
Northern white-cedar	2%	38%	100%
Paper birch	1%	63%	100%
Pignut hickory	5%	99%	100%
Quaking aspen	15%	51%	100%
Red maple	1%	34%	100%
Red pine	5%	38%	100%
Red spruce	3%	73%	100%
Scarlet oak	5%	100%	100%
Sugar maple	5%	52%	100%
Sweet birch	2%	96%	100%
White ash	3%	34%	100%
White oak	0%	40%	100%
Yellow birch	2%	52%	100%
Yellow poplar	0%	99%	100%

Table S2. Proportion of stems, by species and scenario, that experienced annual temperature and precipitation greater than or less than the range of climate conditions used by Thomas et al. (2010) to develop the species-specific growth and survival relationships.

Species	Precipitation				Temperature			
	Constant	RCP2.6	RCP6.0	RCP8.5	Constant	RCP2.6	RCP6.0	RCP8.5
American basswood	1%	1%	2%	3%	0%	0%	0%	0%
American beech	30%	39%	53%	66%	65%	52%	43%	76%
Balsam fir	4%	4%	6%	12%	1%	0%	27%	77%
Bigtooth aspen	0%	0%	2%	4%	0%	1%	6%	17%
Black cherry	0%	1%	1%	2%	0%	0%	2%	8%
Black oak	0%	0%	0%	0%	0%	3%	26%	53%
Chestnut oak	0%	0%	1%	1%	0%	1%	26%	64%
Eastern hemlock	0%	0%	0%	1%	0%	0%	0%	0%
Eastern white pine	0%	0%	0%	1%	0%	1%	6%	20%
Northern red oak	0%	1%	1%	2%	0%	0%	5%	16%
Northern white-cedar	0%	0%	1%	2%	0%	3%	78%	100%
Paper birch	1%	1%	1%	1%	0%	2%	28%	69%
Pignut hickory	0%	0%	1%	1%	0%	6%	46%	75%
Quaking aspen	0%	0%	0%	1%	0%	0%	0%	8%
Red maple	0%	0%	0%	0%	0%	0%	5%	14%
Red pine	0%	0%	1%	2%	3%	1%	3%	25%
Red spruce	0%	0%	0%	1%	0%	1%	21%	78%
Scarlet oak	0%	1%	1%	2%	0%	0%	13%	44%
Sugar maple	0%	0%	0%	0%	0%	1%	10%	20%
Sweet birch	1%	1%	2%	2%	0%	5%	22%	60%
White ash	0%	0%	0%	0%	0%	1%	10%	26%
White oak	0%	0%	0%	1%	0%	0%	22%	56%
Yellow birch	0%	0%	0%	0%	1%	1%	6%	28%
Yellow poplar	1%	1%	1%	2%	0%	3%	37%	83%

Table S3. Species-specific uses, values, and services provided by the 24 northeastern U.S. tree species reported by Thomas et al., (2010) (from http://www.fs.fed.us/database/feis/plants/tree/acesac/all.html)

Species	Species Uses and Values
Abies balsamea	Major food for moose during winter (Peek, 1974) as well as caribou, white-tailed deer, and ruffed grouse.
	Used primarily for pulpwood and lumber for light frame construction including log cabins. Paneling and crates (Bakuzis and Hanset, 1965; Frank, 1990).
	Popular Christmas tree, but grown on plantations for this use. Christmas wreathes and souvenir pillows (Frank, 1990).
	Bark blisters contain oleoresin, which is used in the optics industry a medium for mounting microscope specimens and as a cement for various parts of optical systems (Frank, 1990).
Acer rubrum	Red maple is an important source of sawtimber and pulpwood (Haag et al., 1989) but is often overlooked as a wood resource (Braiewa et al., 1985). The wood is used for furniture, veneer, pallets, cabinetry, plywood, barrels, crates, flooring, and railroad ties (Duncan and Duncan, 1987; Hosie, 1969; Lees, 1981). Can be used for syrup.
	Tolerant of flooding and water-logged soils (Ahlgren and Hansen, 1957; Arthur et al. 1981).
Acer saccharum	Commonly used to make furniture, paneling, flooring, and veneer (Duncan and Duncan, 1988; Kriebel and Gabriel, 1969). It is also used for gunstocks, tool handles, plywood dies, cutting blocks, woodenware, novelty products, sporting goods, bowling pins, and musical instruments (Curtis, 1959; Hosie, 1969; Kriebel and Gabriel, 1969). Source of maple syrup.
	Browsed by moose, white-tailed deer, and snowshoe hare.
	Often associated with stream terraces, streambanks, valleys, canyons, ravines, and wooded natural levees (Adams and Anderson, 1980; Clewell, 1985; Godfrey, 1988; Stephens, 1973).
	Sap can be made into an edible syrup, tea can also be made from bark (Godman and Tubbs, 1973). Ethanol is another product (Hosie, 1969).

Species	Species Uses and Values
Betula papyrifera	Paper birch wood is used commercially for veneer, plywood, and pulpwood. It is easily worked and takes finishes and stains readily. Furniture, cabinets, and numerous specialty items are made from paper birch lumber (Braun, 1961; Safford et al., 1990).<br>Leaves turn yellow to orange or deep red in the fall (Braun, 1961).
	Sugar maple is commonly browsed by white-tailed deer, moose, and snowshoe hare (Godman and Tubbs, 1973; Newton et al., 1989; Telfer, 1972). Importance for hunting.
	Tree chips are used for pulp and paper manufacture, reconstituted uses, and fuel. It is commonly used as fireplace and wood stove fuel (Braun, 1961; Safford et al., 1990).
	Paper birch is useful for long-term revegetation and soil stabilization of severely disturbed sites. It is used to reclaim coal, lignite, rock phosphate, slate, gold, oil-shale, bauxite, and other mine spoils (Safford et al., 1990, Watson et al., 1980).
	Paper birch's graceful form and attractive bark make it a popular landscape plant (Safford et al., 1990). The sap is made into syrup, wine, beer, and medicinal tonics. Currently only a few small sugaring operations in Alaska utilize paper birch (Safford et al., 1990)
	Native Americans made paper birch bark into baskets, storage containers, mats, baby carriers, moose and bird calls, torches, household utensils, and canoes (Holloway and Alexander, 1990). The strong and flexible wood was made into spears, bows, arrows, snowshoes, sleds, and other items (Holloway and Alexander, 1990).
Carya glabra	Pignut hickory wood is heavy, hard, strong, tough, and elastic (Ontario Department of Lands and Forests, 1953; Vines, 1960). Early uses included broom handles, skis, wagon wheels and, early automobile parts (Ontario Department of Lands and Forests, 1953; Vines, 1960). Sporting goods, agricultural implements, and tool handles are made from the wood of pignut hickory (Hosie, 1969; Ontario Department of Lands and Forests, 1953; Vines, 1960). Specialty products include shuttle blocks, mallets, and mauls (Smalley, 1990).
	Hickory nuts a moderate source of food for wildlife especially squirrels and chipmunks (Smalley, 1990).
	It recolonizes abandoned strip mines in Maryland and West Virginia (Hardt and Forman, 1989).
	Nuts of pignut hickory are large and edible (Monk, 1968) and in some areas are grown commercially, although they are of minor importance when compared to shagbark hickory nuts (Graney, 1990).

Species	Species Uses and Values
	Pignut hickory is used as a shade tree throughout much of its range (Smalley, 1990).
Fraxinus americana	The wood of white ash is economically important due to its strength, hardness, weight, and shock resistance (Hosie, 1969). It is second only to hickory (Carya spp.) for use in the production of tool handles. Nearly all wooden baseball bats are made from white ash (Ganser and Widmann, 1990). The wood is also used in furniture, antique vehicle parts, railroad cars and ties, canoe paddles, snowshoes (Millers et al., 1989), boats, doors, and cabinets (Vines, 1960).
	Good forage for birds and small mammals (Schlesinger, 1990). Provide habitat for cavity-nesters (DeGraaf and Shigo, 1985).
	The juice from the leaves of white ash can be applied topically to mosquito bites for relief of swelling and itching (Hosie, 1969).
	White ash has a specialized use as a prophylactic measure for snake bite. If one carries the crushed leaves in his/her pockets the odor has been "proved" offensive to rattlesnakes (Schlesinger, 1990).
	Open-grown white ash is useful as a shade and ornamental tree (Hosie, 1969).
	White ash has a strong affinity for soils high in nitrogen and calcium (Schlesinger, 1990).
Liriodendron tulipifera	Yellow-poplar wood is used for construction grade lumber and plywood (Beck, 1990). It has straight grain, little shrinkage, and excellent gluing qualities (Beck and Della-Bianca, 1981). In the past it was used for carriage bodies, shingles, saddle frames, and interior finish wood. It is currently used for cabinets, veneer, furniture, and pulp (Beck and Della-Bianca, 1981).
	Only fair value as a fuelwood but good value as kindling (Carey and Gill, 1980).
	Livestock and wildlife both browse on this tree (Beck, 1990; Michael, 1988).
	Yellow-poplar has been valued as an ornamental since 1663. The tulip like flowers and leaves are aesthetically pleasing (Beck and Della-Bianca, 1981). The flowers are also valuable nectar producers. The flowers from a 20-year-old tree produce enough nectar to yield 4 pounds (1.8 kg) of honey (Beck, 1990).
	Yellow-poplar was used medicinally in the late 1800s: a heart stimulant was extracted from the inner bark of the root (Hosie, 1969), and a tonic for treating rheumatism and dyspepsia was extracted from stem bark (Lamson, 1983).
Pinus resinosa	Gradual climate warming is predicted to temporarily increase red pine abundance but eventually lead to decline (He et al., 2002)

Species	Species Uses and Values
Pinus resinosa	Provides habitat for birds and small mammals (Benzie, 1977; Rudolf, 1990; Sims et al., 1990)
	Used as an ornamental (Duncan and Duncan, 1988)
	Red pine is a very important source of wood products (Anonymous, 1990; Duncan and Duncan, 1988; Eyre and Zehngraff, 1948; Farrar, 1995). It is used for lumber, pilings, poles, cabin logs, railway ties, posts, mine timbers, box boards, pulpwood, and fuel (Benzie, 1977; Farrar, 1995; Rudolf, 1990; Sims et al., 1990).
Pinus strobus	Eastern white pine is a valuable timber species in the eastern United States and Canada. The soft wood is of medium strength, easily worked, and stains and finishes well. It is used for doors, moldings, trim, siding, paneling, cabinet work, and furniture (Hosie, 1969; Wendel and Smith, 1990).
	Eastern white pine provides food and habitat for numerous wildlife species. Songbirds and small mammals eat eastern white pine seeds. Snowshoe hares, white-tailed deer, and cottontails browse the foliage; the bark is eaten by various mammals (Wendel and Smith, 1990).
	Eastern white pine is used extensively for stabilizing strip-mine spoils, especially in northern Appalachian coal fields (Torbert et al., 1988; Vogel, 1981)
	The frequency of eastern white pine is lower in today's forests than in presettlement forests. Eastern white pine was heavily logged in the 1800s in the north-central United States (Nowacki and Abrams, 1992).
Populus grandidentata	It is primarily used for pulp, but is also used to make particle board and structural panels. Minor uses include log homes, pallets, boxes, match splints, chopsticks, hockey stick components, and ladders (Morley and Balatinecz, 1993; Perala and Carpenter, 1985).
	Bigtooth aspen bark is pelletized for fuel and supplemental cattle feed (Laidly, 1990).
	Bigtooth aspen provides food and cover for wildlife. Moose and white-tailed deer browse bigtooth aspen (Allen et al., 1987; Stormer and Bauer, 1980). Beaver eat bark, leaves, twigs, and branches (Laidly, 1990).

Species	Species Uses and Values
Populus tremuloides	Quaking aspen is one of the most important timber trees in the East. Its wood is used primarily for particleboard, especially waferboard and oriented strandboard, and for pulp. In the Great Lakes States, quaking aspen fibers are well suited for making fine paper. Some quaking aspen is used for lumber. Quaking aspen lumber is used for making boxes, crates, pallets, and furniture. A small but growing volume is made into studs. Quaking aspen wood is little used in the West, except in Colorado, where it is used for pulp and particleboard (Perala and Carpenter, 1985). Specialty products from quaking aspen wood include excelsior, matchsticks, and tongue depressors.
	Quaking aspen pellets are used for fuel (Perala and Carpenter, 1985; Youngquist and Spelter, 1990).
	Quaking aspen forests provide important breeding, foraging, and resting habitat for a variety of birds and mammals. It is especially valuable during fall and winter, when protein levels are high relative to other browse species (Tew, 1970).
	Aspens (Trepidae) are unique in their ability to stabilize soil and watersheds. Fire-killed stands are promptly revegetated by root sprouts (suckers). The trees produce abundant litter that contains more nitrogen, phosphorus, potash, and calcium than leaf litter of most other hardwoods. The litter decays rapidly, forming a nutrient-rich humus that may amount to 25 tons per acre (oven-dry basis). The humus reduces runoff and aids in percolation and recharge of ground water. Soil under quaking aspen thaws faster and infiltrates snow more rapidly than soil under conifers (Brinkman and Roe, 1975).
	Mountain slopes covered by quaking aspen provide high yields of good-quality water. Quaking aspen intercepts less snow than conifers, so snowpack is often greater under quaking aspen (DeByle, 1985).
	Quaking aspen is valued for its aesthetic qualities at all times of the year. The yellow, orange, and red foliage of autumn particularly enhances recreational value of quaking aspen sites (Johnson et al., 1985).
	Quaking aspen is widely used in ornamental landscaping (Johnson et al., 1985).
Prunus serotina	Black cherry is an important commercial tree. The rich reddish-brown wood is strong, hard, and close-grained. It works well and finishes smoothly, making it one of the most valued cabinet and furniture woods in North America (Vines, 1960). Black cherry wood is also used for paneling, interior trim, veneers, handles, crafts, toys, and scientific instruments (Duncan and Duncan, 1988; Van Dersal, 1938). Black cherry's commercial range, where large numbers of high-quality trees are found, is restricted to the Allegheny Plateau of Pennsylvania, New York, and West Virginia (Marquis, 1990).

Species	Species Uses and Values
	Black cherry leaves, twigs, bark, and seeds are poisonous to livestock. They contain a cyanogenic glycoside that breaks down during digestion into hydrocyanic acid (Stephens, 1980).
	Black cherry fruits are important mast for numerous species of birds and mammals (Chapman and Bessette, 1990; Martin et al., 1951; Morden-Moore and Wilson, 1982; Van Dersal, 1938).
	Black cherry bark was used historically in the Appalachians as a cough remedy, tonic, and sedative. The fruit was also used to flavor rum and brandy. Pitted fruits are edible, and are eaten raw and used in wine and jelly (Marquis, 1990).
Quercus coccinea	Although scarlet oak wood is of inferior grade, it is cut and utilized with other red oaks as red oak lumber (Harlow et al., 1979).
	Scarlet oak acorns are an important food source for numerous upland wildlife species including squirrels, chipmunks, mice, wild turkeys, white-tailed deer, blue jays, and woodpeckers (Johnson, 1990).
	Scarlet oak is widely planted in the United States and Europe as a shade tree and ornamental. It has brilliant red foliage in autumn (Johnson, 1990).
Quercus prinus	Chestnut oak wood is cut and utilized s white oak lumber (McQuilkin, 1990).
	Good crops of chestnut oak acorns are infrequent, but when available the acorns are eaten by numerous upland wildlife species (Little et al., 1958).
	Chestnut oak is one of the two most preferred host species of the introduced gypsy moth, which defoliates trees (McQuilkin, 1990).
Quercus rubra	Northern red oak is an important source of hardwood lumber (Chapman and Bessette, 1990; Maeglin, 1974). Its wood is heavy, hard, strong, coarse-grained, and at least moderately durable (Ontario Department of Lands and Forests, 1953). When properly dried and treated, oak wood glues well, machines very well, and accepts a variety of finishes (Moser, 1971). The wood of northern red oak has been used to make railroad ties, fence posts, veneer, furniture, cabinets, paneling, flooring, caskets, and pulpwood (Millers et al., 1989; Ontario Department of Lands and Forests, 1953).
	Northern red oak has a high fuel value and is an excellent firewood (Millers et al., 1989).
	Acorns of the northern red oak are an important food source for small mammals and birds (Pekins and Mautz, 1988; Sork et al., 1983; Van Dersal, 1940)

Species	Species Uses and Values
	The acorns of many species of oak (Quercus spp.) were traditionally an important food source for Native American peoples (Van Dersal, 1938).
	Acorns of red oak were leached with ashes to remove bitter tannins and then used in various foods by many Native American peoples. Preparations made from the bark were used to treat bowel problems (Gilmore, 1919).
	Northern red oak was first cultivated in 1724 (Olson, 1974) and is a popular ornamental shade tree in eastern North America and in parts of Europe (Hosie, 1969; Sander, 1990).
Thuja occidentalis	The wood of northern whitecedar is resistant to decay. It is used for products that come in contact with water and soil, such as fence posts, shingles, paneling, and boats (Hosie, 1969; Johnston, 1990). Northern whitecedar logs are especially popular to use for log cabins because the wood has good insulating qualities (Lanasa, 1989). It is also used for kraft pulp and particle board (Johnston, 1990).
	Northern whitecedar provides food and shelter for wildlife. White-tailed deer, snowshoe hares, and porcupines heavily browse the foliage (Johnston, 1990). Northern whitecedar is one of the best winter browse species for white-tailed deer in the northern Lake States, and it is often over browsed (Aldous, 1952).
	Northern whitecedar is widely planted as an ornamental. Northern whitecedar leaf oil is distilled from boughs and used for perfume and medicines. The foliage is rich in vitamin C; Native Americans and early European explorers used it to treat scurvy (Johnston, 1990).
	Because of its long life span, northern whitecedar is a valuable species for dendroclimatic research (Archambault and Bergeron, 1992).
Tilia americana	Basswood wood is soft and light; it is valued for hand carving and has many other uses including cooperage, boxes, veneer, excelsior, and pulp (Collingwood, 1937; Crow, 1990). Basswood is economically important for timber, especially in the Great Lakes states (Crow, 1990).
	Carey and Gill (1980) rated basswood as fair (their lowest rating) for firewood.
	Basswood is preferred browse for white-tailed deer. Basswood flowers are visited by honeybees for nectar (Crow, 1990). The easily decayed wood produces a disproportionate number of cavities, which are used by cavity-nesting animals including wood ducks (Dugger and Fredrickson, 1992), pileated woodpeckers (Hardin and Evans, 1977), other birds, and small mammals (Carey, 1983). Carey and Gill (1980) rated basswood as fair (their lowest rating) for all wildlife.

Species	Species Uses and Values
Picea rubens	Red spruce is one of the more important timber species in the northeastern United States. The wood is light in weight, straight grained, and resilient. It is used for paper, construction lumber, and is highly preferred for musical instruments (Blum, 1990; Hart, 1959).
	Spruce grouse browse the leaves and twigs of red spruce (Pielou, 1988). Mice and voles consume and store significant amounts of spruce seeds, preferring red and white spruce to balsam fir (Abbot, 1962). Birds (particularly crossbills or grosbeaks) will clip the terminal buds of young spruce, as will porcupines, bears, snowshoe hares, and, rarely, deer (Blum, 1977; McIntosh and Hurley, 1964; Stickney, 1989). Red squirrels clip twigs and terminal buds and also eat reproductive and vegetative buds (Blum, 1977; Safford, 1974).
	Red spruce gum was formerly collected and processed for chewing gum (Hart, 1959).

Table S4. Average timber stumpage prices by species and state for 2015 or most recently reported prior year ($/MBF)

Species:	CT/MA/RI	IL	IN	KY	ME	MI	NH	NY	OH	PA	VT	WV	WI	Number of States with Reported Prices	Mean Value	State-Biomass-Weighted Average Price
Black Cherry	$151	$350	$796	$480		$364		$526	$499	$590		$371	$282	10	$441	$504
Sugar Maple	$186	$298	$879	$647	$286	$677	$243	$534	$547	$394	$333	$280	$341	13	$434	$438
Chestnut Oak				$654				$208						2	$431	$560
Northern Red Oak			$710	$584	$273	$292		$481	$525	$481	$319	$235	$314	10	$421	$390
White Oak	$105	$513	$768	$699	$147	$95	$270	$239	$570	$392		$240	$213	12	$354	$429
Black Oak		$225		$463										2	$344	$398
Pignut Hickory			$732	$413				$119	$249			$107		5	$324	$311
White Ash	$106	$238	$614	$455	$176	$119	$114	$298	$345	$293	$236	$166	$181	13	$257	$259
Red Maple	$37	$225	$710	$366	$148	$216	$63	$213	$296	$239	$136	$183	$200	13	$233	$188
American Basswood		$158	$392	$288		$163		$113	$185				$143	7	$206	$163
Yellow Birch	$61				$219	$282	$166	$188			$263		$219	7	$200	$209
Yellow Poplar	$36	$200	$200	$377				$122	$268	$224		$152		8	$197	$244
Eastern White Pine	$93			$150	$161	$123	$135	$91		$78	$116		$124	9	$119	$118
American Beech	$22	$93	$450		$49	$93		$57			$55			7	$117	$59
Northern White Cedar					$94						$103		$118	3	$105	$99
Balsam Fir					$152		$105						$54	3	$104	$114
Bigtooth Aspen					$115	$92								2	$103	$94
Red Pine	$48				$67	$247	$42	$79					$85	6	$95	$172
Red Spruce	$34				$152		$105	$91			$99		$84	6	$94	$122
Eastern Hemlock	$32			$206	$73		$42	$50		$70	$58		$145	8	$84	$62
Paper Birch	$33					$118	$79							3	$76	$68
Quaking Aspen						$65		$43					$50	3	$53	$57
Scarlet Oak														0		
Sweet Birch														0		

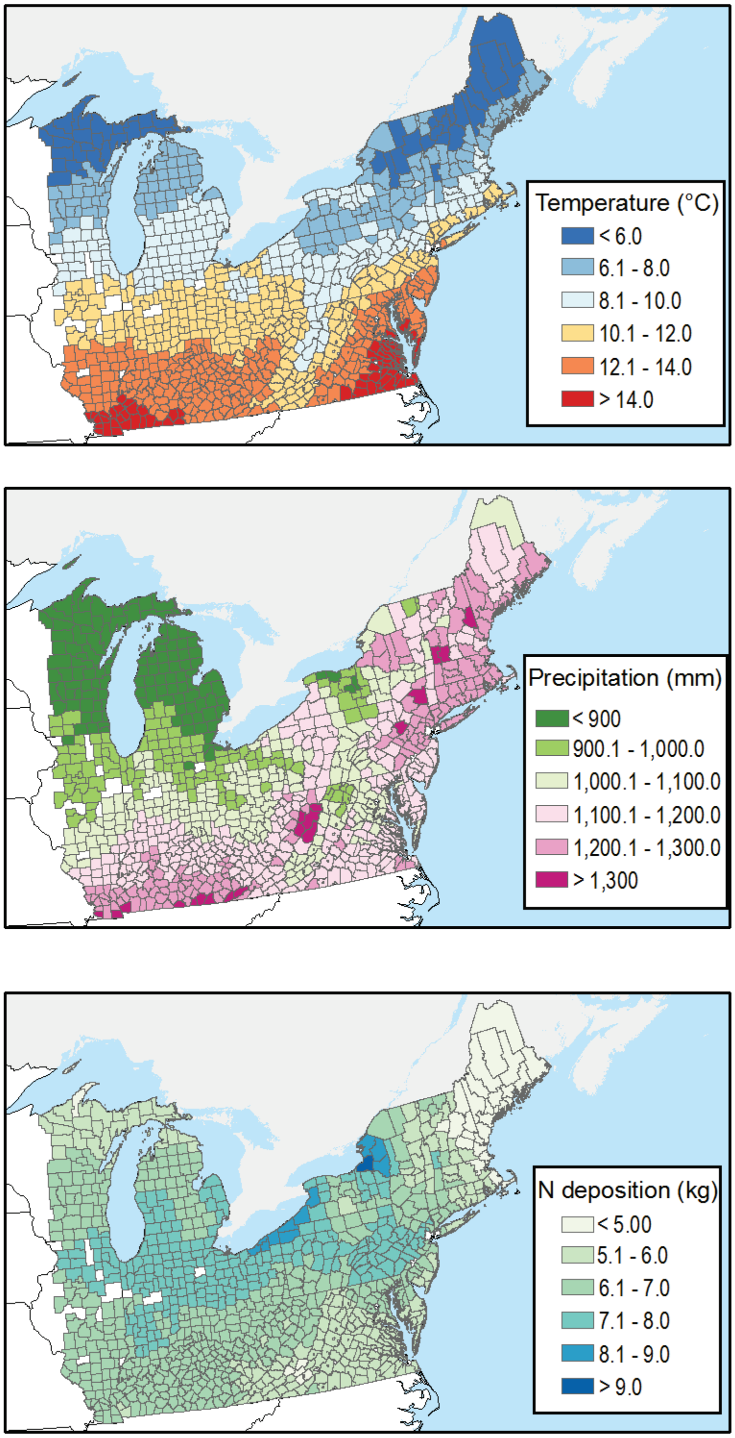

Figure S1. Baseline conditions: county-level average annual temperature, average annual precipitation, and average N deposition in 2005.

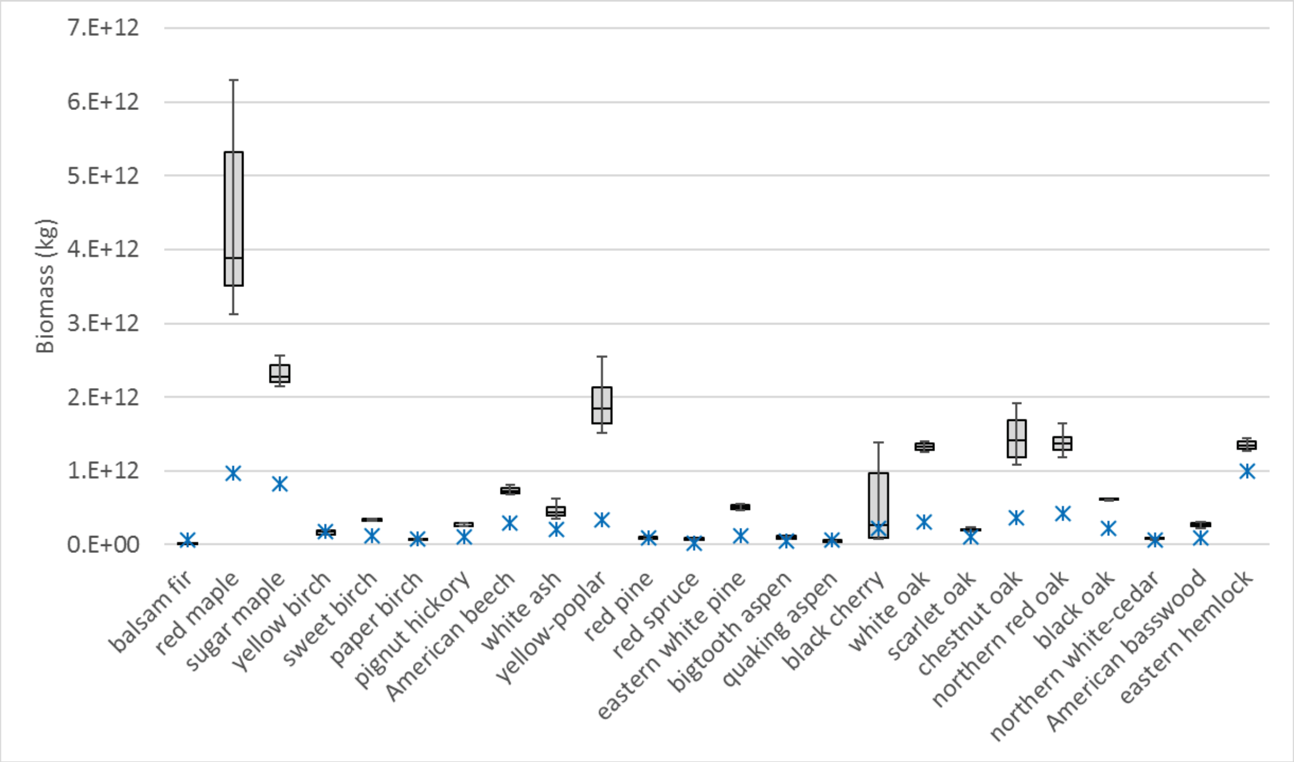

Figure S2. Ranges of species-specific biomass in 2100 across the twelve N deposition–climate scenarios. Box plots represent the 25th and 75th percentiles and the whiskers represent the 5th and 95th percentiles for the twelve scenarios in 2100. The * associated with each box plot is the biomass of the species in 2005.

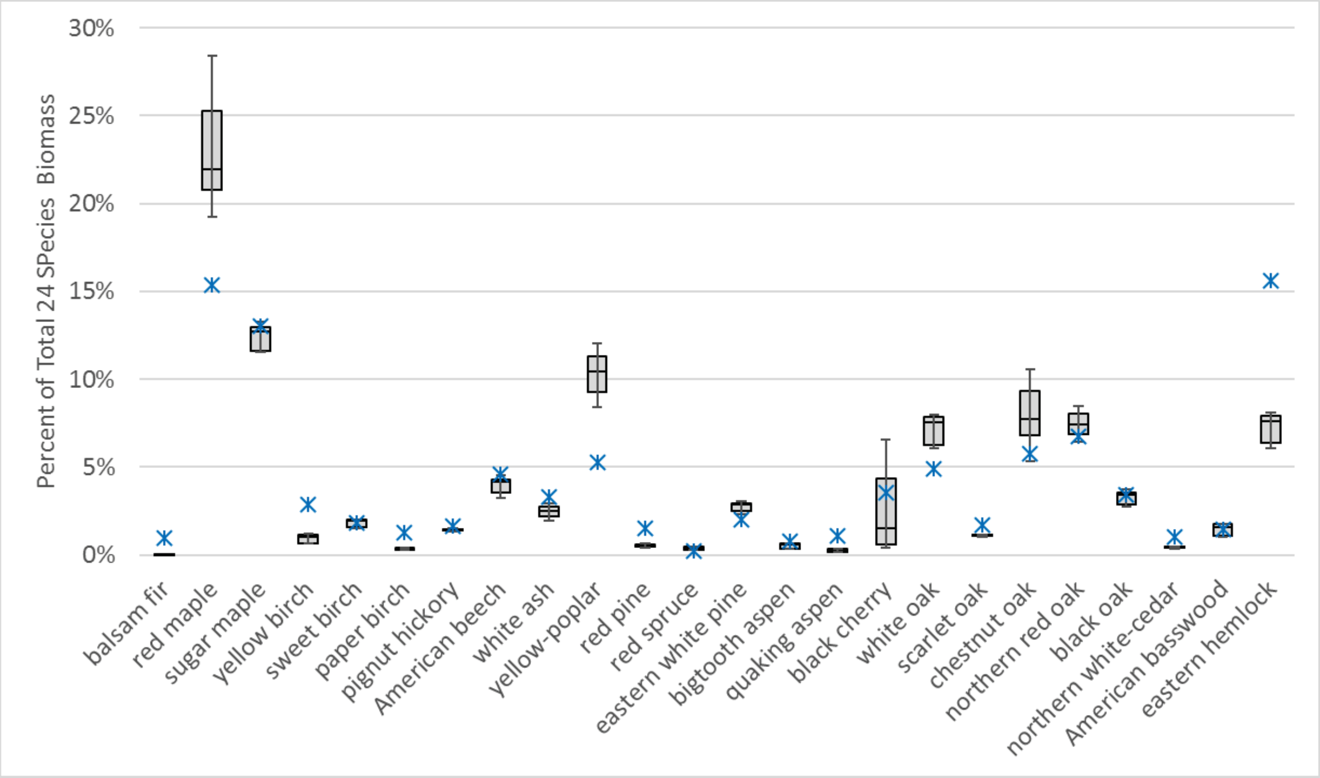

Figure S3. Ranges of species-specific relative biomass (% of total biomass of the 24 species) in 2100 across the twelve N deposition–climate scenarios. Box plots represent the 25th and 75th percentiles and the whiskers represent the 5th and 95th percentiles for the twelve scenarios in 2100. The * associated with each box plot is the relative biomass of the species in 2005.

Figure S4. Maps of county-level relative abundance (first column), change in relative abundance (second column), and contribution to changes in Shannon index (third column) for each tree species. All changes are between the high pollution scenario (RCP 8.5/Constant) and the low pollution scenario (Constant/Return to PE).

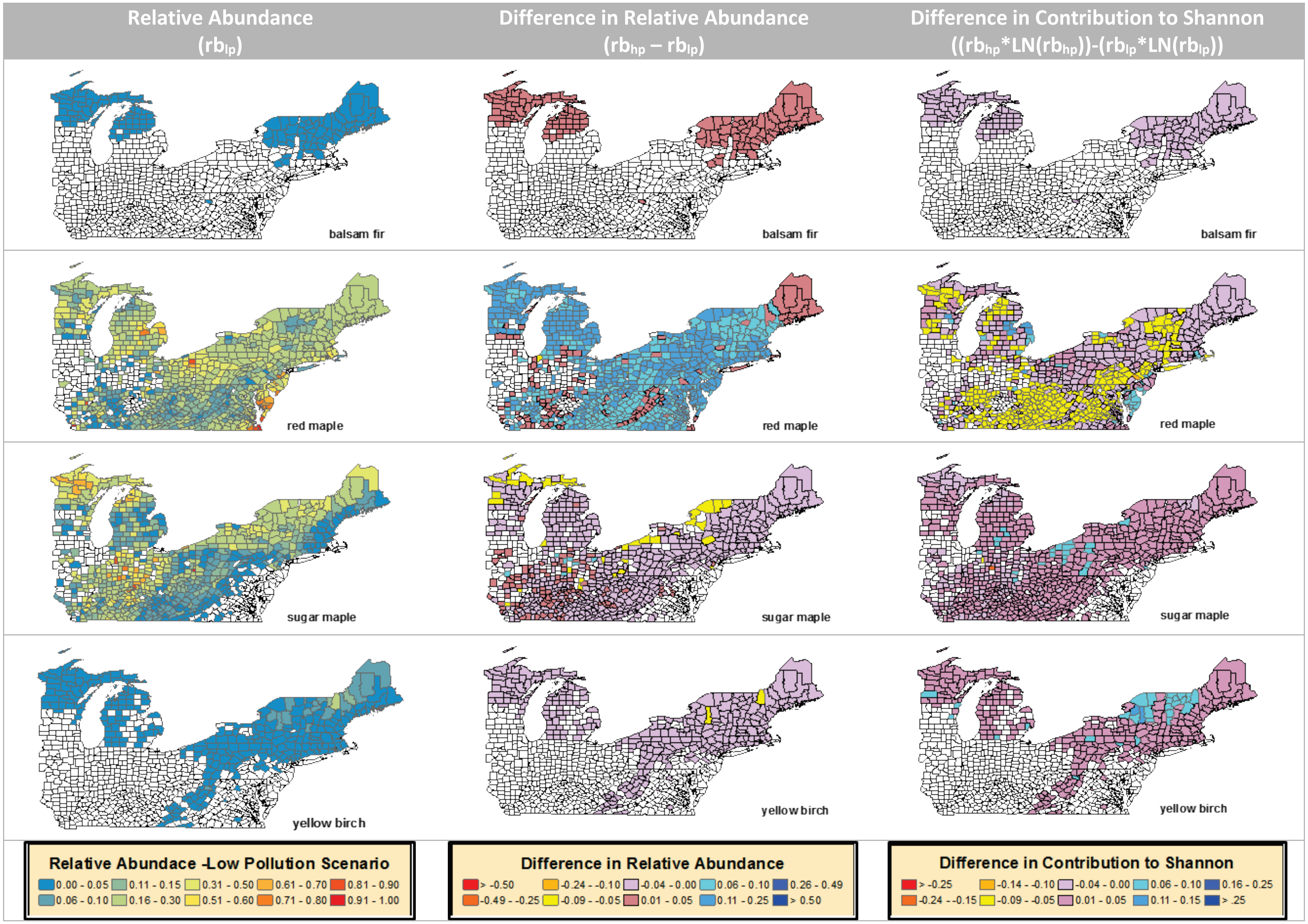

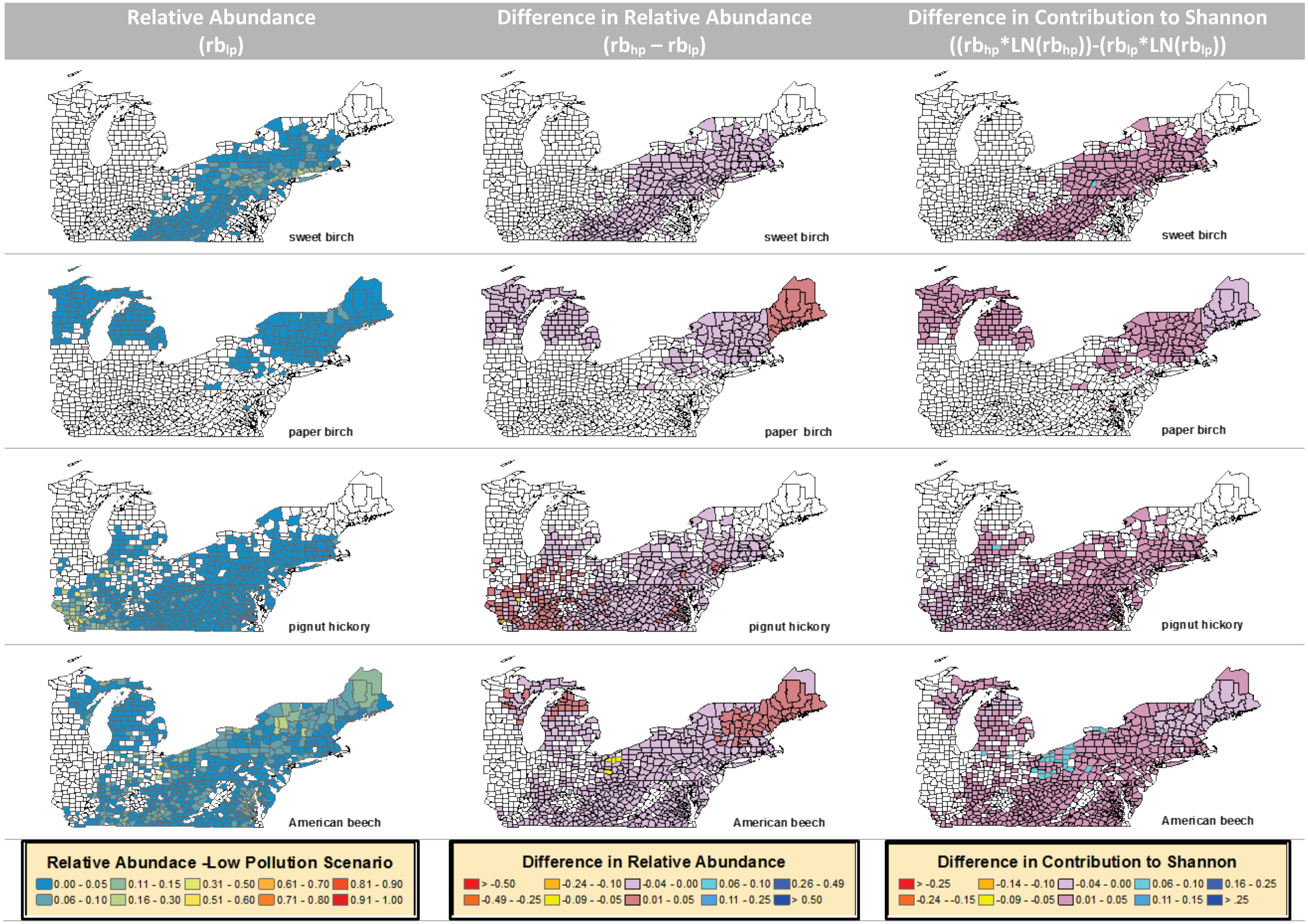
Relative Abundance
(rblp)
Difference in Relative Abundance
(rbhp – rblp)
Difference in Contribution to Shannon
((rbhp*LN(rbhp))-(rblp*LN(rblp))
sweet birch
sweet birch
sweet birch
paper birch
paper birch
paper birch
pignut hickory
pignut hickory
pignut hickory
American beech
American beech
American beech
Relative Abundace -Low Pollution Scenario
0.00 - 0.05
0.06 - 0.10
0.11 - 0.15
0.16 - 0.30
0.31 - 0.50
0.51 - 0.60
0.61 - 0.70
0.71 - 0.80
0.81 - 0.90
0.91 - 1.00
Difference in Relative Abundance
> -0.50
-0.49 - -0.25
-0.24 - -0.10
-0.09 - -0.05
-0.04 - 0.00
0.01 - 0.05
0.06 - 0.10
0.11 - 0.25
0.26 - 0.49
> 0.50
Difference in Contribution to Shannon
> -0.25
-0.24 - -0.15
-0.14 - -0.10
-0.09 - -0.05
-0.04 - 0.00
0.01 - 0.05
0.06 - 0.10
0.11 - 0.15
0.16 - 0.25
> .25

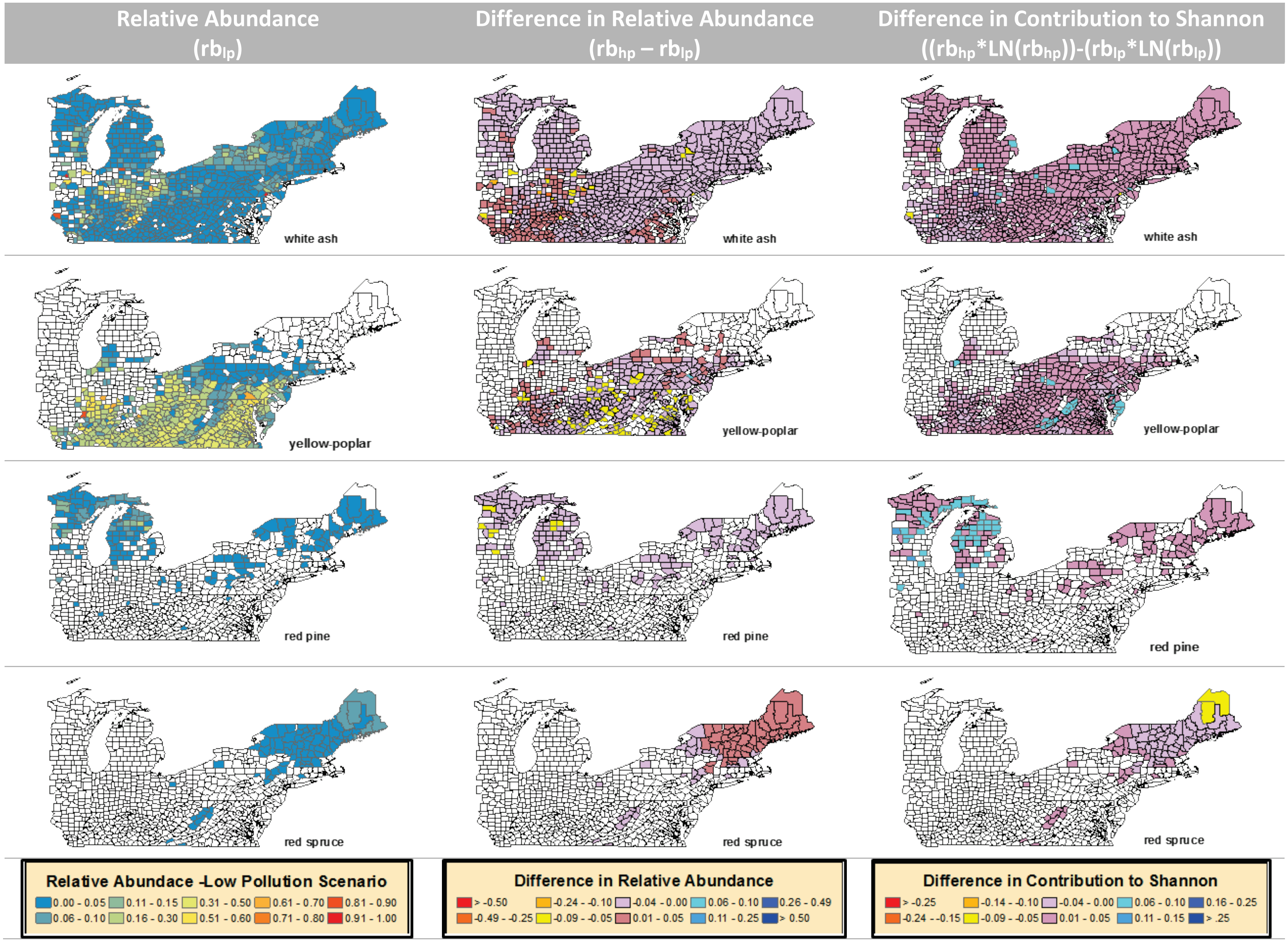
Relative Abundance
(rb_{lp})
Difference in Relative Abundance
($rb_{hp} – rb_{lp}$)
Difference in Contribution to Shannon
($(rb_{hp}*LN(rb_{hp}))-(rb_{lp}*LN(rb_{lp}))$)
white ash
white ash
white ash
yellow-poplar
yellow-poplar
yellow-poplar
red pine
red pine
red pine
red spruce
red spruce
red spruce
Relative Abundace -Low Pollution Scenario
0.00 - 0.05
0.11 - 0.15
0.31 - 0.50
0.61 - 0.70
0.81 - 0.90
0.06 - 0.10
0.16 - 0.30
0.51 - 0.60
0.71 - 0.80
0.91 - 1.00
Difference in Relative Abundance
> -0.50
-0.24 - -0.10
-0.04 - 0.00
0.06 - 0.10
0.26 - 0.49
-0.49 - -0.25
-0.09 - -0.05
0.01 - 0.05
0.11 - 0.25
> 0.50
Difference in Contribution to Shannon
> -0.25
-0.14 - -0.10
-0.04 - 0.00
0.06 - 0.10
0.16 - 0.25
-0.24 - -0.15
-0.09 - -0.05
0.01 - 0.05
0.11 - 0.15
> .25

Relative Abundance (rb_{lp}) | Difference in Relative Abundance ($rb_{hp} – rb_{lp}$) | Difference in Contribution to Shannon ($((rb_{hp}*LN(rb_{hp}))-(rb_{lp}*LN(rb_{lp}))$)

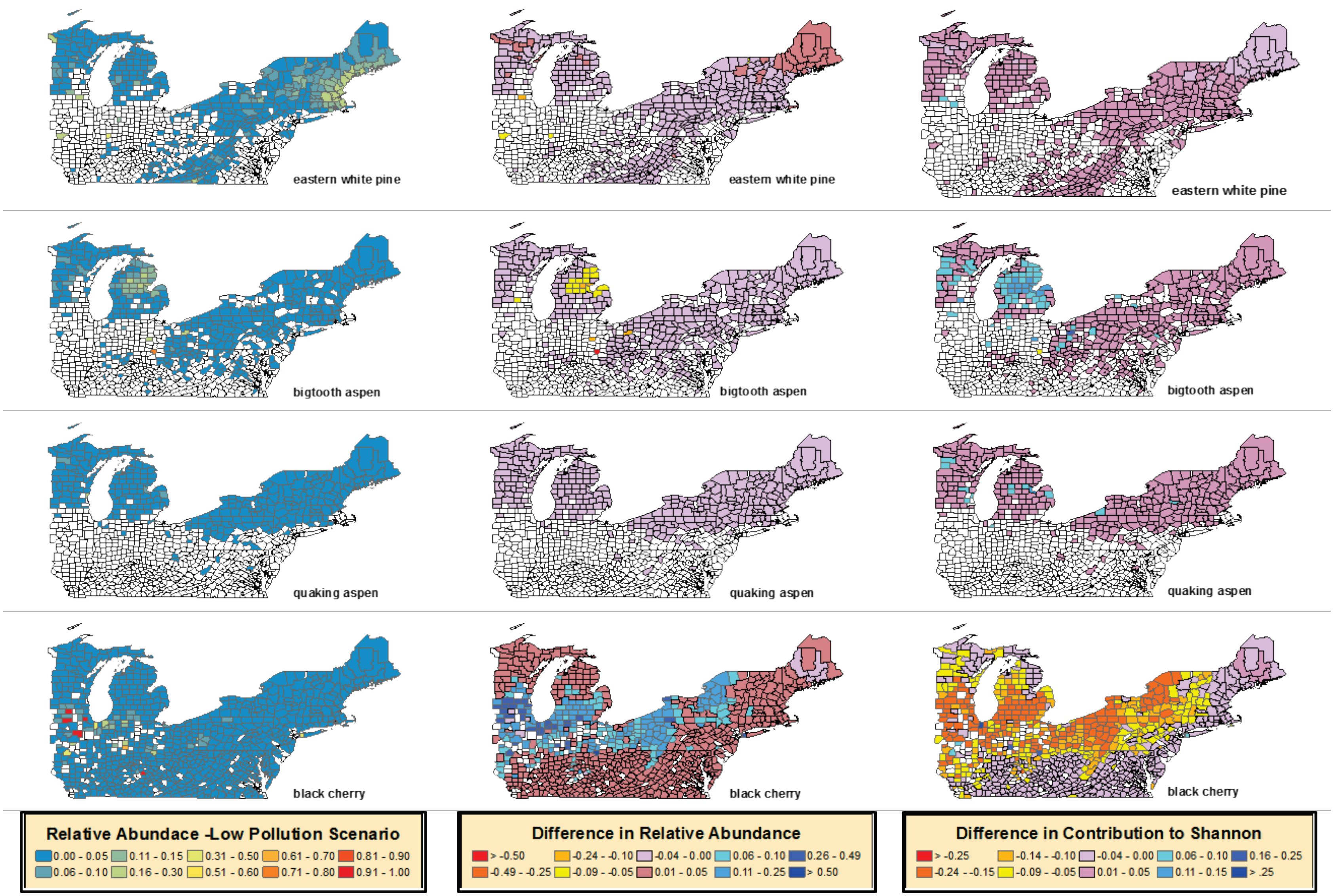

Relative Abundance | Difference in Relative Abundance | Difference in Contribution to Shannon

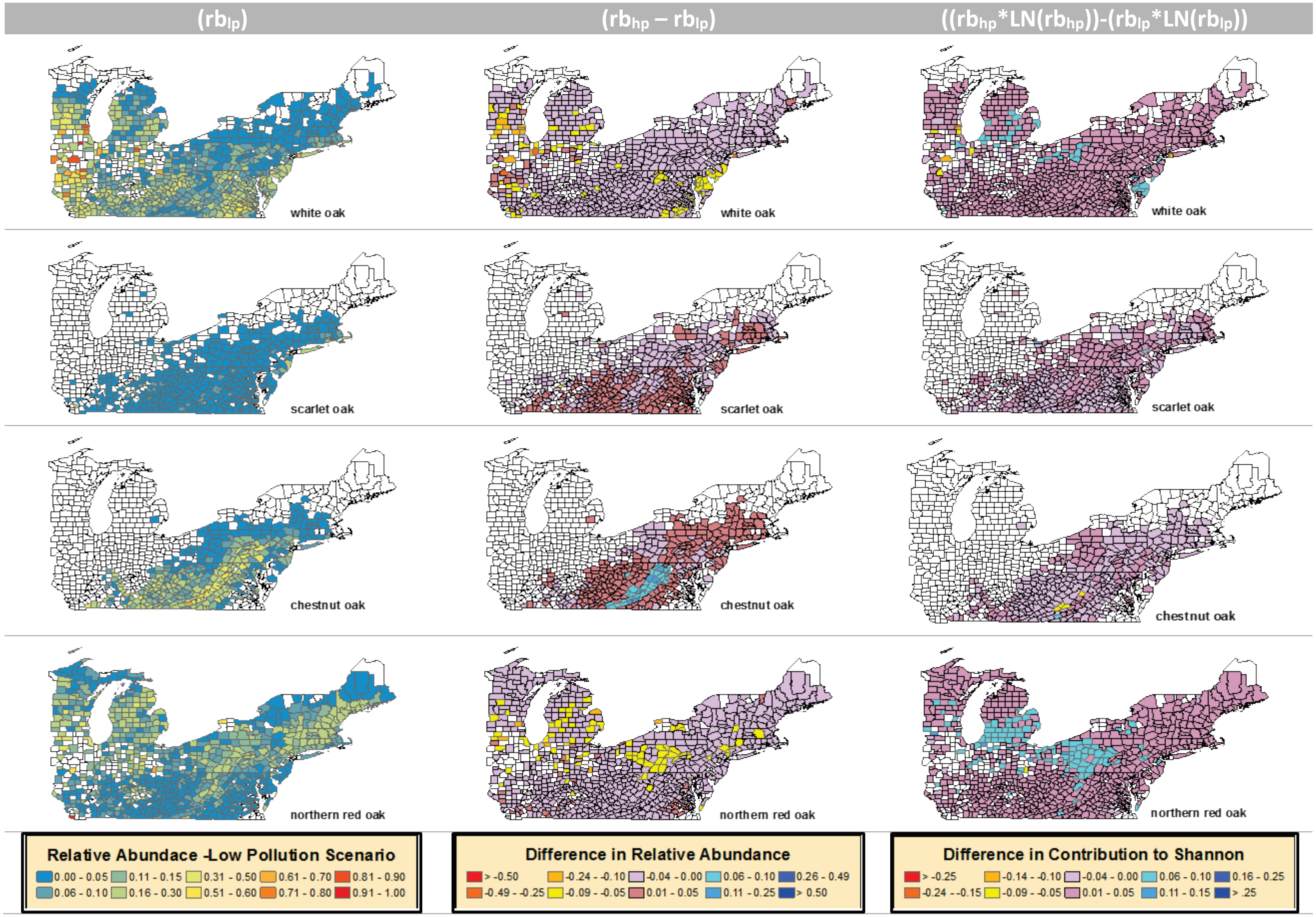

(rb_{lp})
$(rb_{hp} – rb_{lp})$
$((rb_{hp}*LN(rb_{hp}))-(rb_{lp}*LN(rb_{lp}))$
white oak
white oak
white oak
scarlet oak
scarlet oak
scarlet oak
chestnut oak
chestnut oak
chestnut oak
northern red oak
northern red oak
northern red oak
Relative Abundace -Low Pollution Scenario
0.00 - 0.05
0.06 - 0.10
0.11 - 0.15
0.16 - 0.30
0.31 - 0.50
0.51 - 0.60
0.61 - 0.70
0.71 - 0.80
0.81 - 0.90
0.91 - 1.00
Difference in Relative Abundance
> -0.50
-0.49 - -0.25
-0.24 - -0.10
-0.09 - -0.05
-0.04 - 0.00
0.01 - 0.05
0.06 - 0.10
0.11 - 0.25
0.26 - 0.49
> 0.50
Difference in Contribution to Shannon
> -0.25
-0.24 - -0.15
-0.14 - -0.10
-0.09 - -0.05
-0.04 - 0.00
0.01 - 0.05
0.06 - 0.10
0.11 - 0.15
0.16 - 0.25
> .25

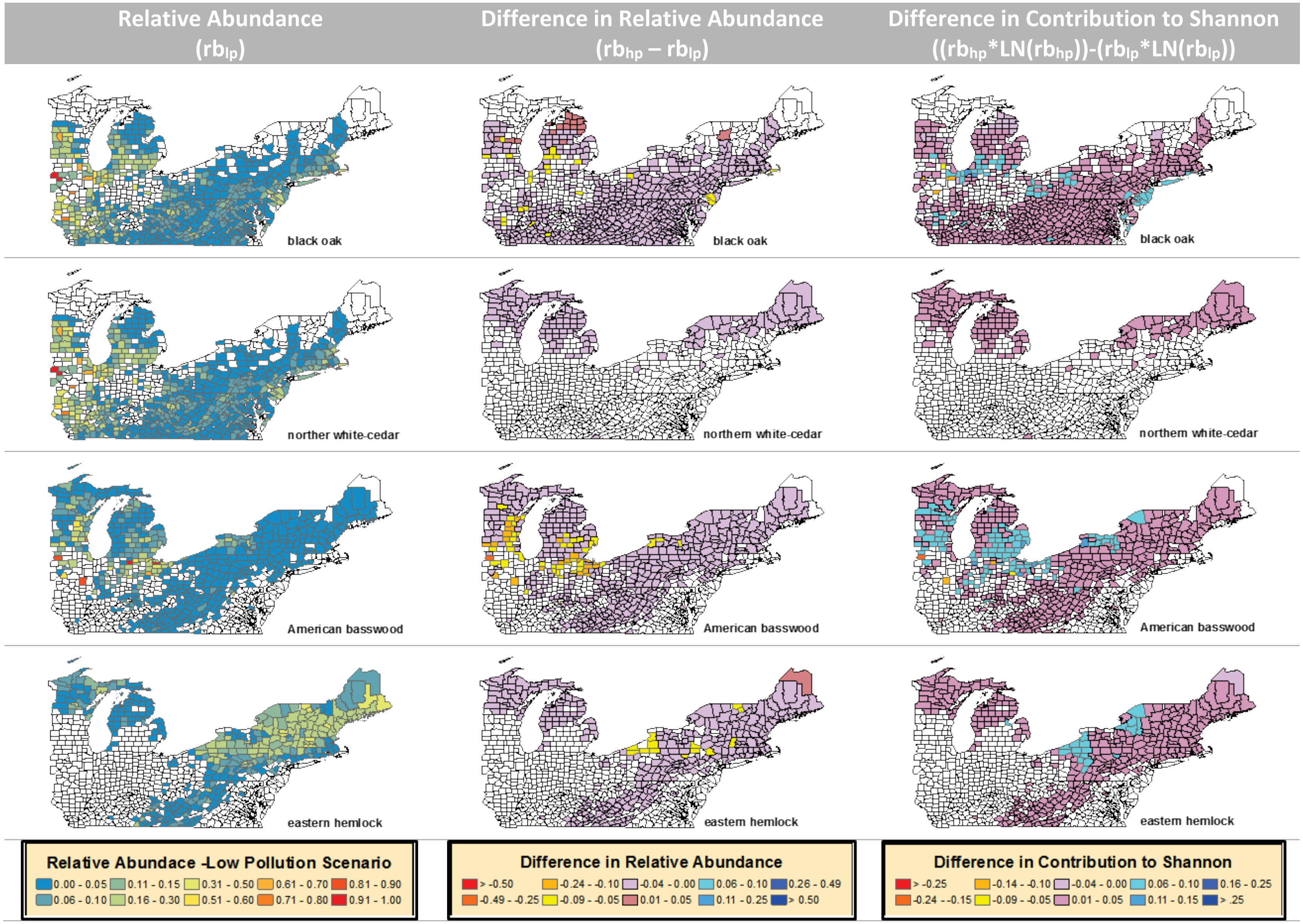
Relative Abundance
(rblp)
Difference in Relative Abundance
(rbhp – rblp)
Difference in Contribution to Shannon
((rbhp*LN(rbhp))-(rblp*LN(rblp))
black oak
black oak
black oak
norther white-cedar
northern white-cedar
northern white-cedar
American basswood
American basswood
American basswood
eastern hemlock
eastern hemlock
eastern hemlock
Relative Abundace -Low Pollution Scenario
0.00 - 0.05
0.06 - 0.10
0.11 - 0.15
0.16 - 0.30
0.31 - 0.50
0.51 - 0.60
0.61 - 0.70
0.71 - 0.80
0.81 - 0.90
0.91 - 1.00
Difference in Relative Abundance
> -0.50
-0.49 - -0.25
-0.24 - -0.10
-0.09 - -0.05
-0.04 - 0.00
0.01 - 0.05
0.06 - 0.10
0.11 - 0.25
0.26 - 0.49
> 0.50
Difference in Contribution to Shannon
> -0.25
-0.24 - -0.15
-0.14 - -0.10
-0.09 - -0.05
-0.04 - 0.00
0.01 - 0.05
0.06 - 0.10
0.11 - 0.15
0.16 - 0.25
> .25

References

Abbott, H.G. 1962. Tree seed preferences of mice and voles in the Northeast. Journal of Forestry. 60: 97-99.

Hart, A.C. 1959. Silvical characteristics of red spruce. Paper No. 124. Durham, NH: U.S. Department of Agriculture, Forest Service, Northeastern Forest Experiment Station. 18 p

McIntosh, R.P.; Hurley, R. T. 1964. The spruce-fir forest of the Catskill Mountains. Ecology. 45(2): 314-326.

Pielou, E.C. 1988. The world of northern evergreens. Ithaca, NY: Cornell University Press. 174 p

Blum, B.M. 1977. Animal damage to young spruce and fir in Maine. Res. Note NE-321. Upper Darby, PA: U.S. Department of Agriculture, Forest Service, Northeastern Forest Experiment Station. 4 p.

Safford, L.O. 1974. Picea A. Dietr. spruce. In: Schopmeyer, C. S., ed. Seeds of woody plants in the United States. Agric. Handb. 450. Washington, DC: U.S. Department of Agriculture, Forest Service: 587-597.

Stickney, P.F. 1989. Seral origin of species originating in northern Rocky Mountain forests. Unpublished draft on file at: U.S. Department of Agriculture, Forest Service, Intermountain Research Station, Fire Sciences Laboratory, Missoula, MT; RWU 4403 files. 7 p.

Blum, B.M. 1990. Picea rubens Sarg. red spruce. In: Burns, Russell M.; Honkala, Barbara H., technical coordinators. Silvics of North America. Volume 1. Conifers. Agric. Handb. 654. Washington, DC: U.S. Department of Agriculture, Forest Service: 250-259.

Adams, D.E., and Anderson, R.C. 1980. Species response to a moisture gradient in central Illinois forests. American Journal of Botany. 67(3): 381-392.

Ahlgren, C.E. and Hansen, H.L. 1957. Some effects of temporary flooding on coniferous trees. Forestry. 55(9): 647-650.

Aldous, S.E. 1952. Deer browse clipping study in the Lake States Region. Journal of Wildlife Management. 16(4): 401-409.

Allen, A.W., Jordan, P.A., and Terrell, J.W. 1987. Habitat suitability index models: moose, Lake Superior region. Biol. Rep. 82 (10.155). Washington, DC: U.S. Department of the Interior, Fish and Wildlife Service. 47 p.

Anon. 1990. Red pine decline linked to insects, fungi. Northern Logger. 38(7): 3.

Archambault, S., and Bergeron, Y. 1992. An 802-year tree-ring chronology from the Quebec boreal forest. Canadian Journal of Forest Research. 22: 674-682.

Arthur, J.J.; Leone, I. A.; Flower, F. B. 1981. Flooding and landfill gas effects on red and sugar maples. Journal of Environmental Quality. 10(4): 431-433.

Bakuzis, E. V.; Hansen, H. L.; with contrib. by Kaufert, F. H.; Lawrence, D. B.; Duncan, D. P.; [and others]. 1965. Balsam fir, Abies balsamea (Linnaeus) Miller; a monographic review. Minneapolis, MN: The University of Minnesota Press. 445 p.

Beck, D.E. 1990. Liriodendron tulipifera L. yellow poplar. In: Burns, Russell M.; Honkala, Barbara H., technical coordinators. Silvics of North America. Volume 2. Hardwoods. Agric. Handb. 654. Washington, DC: U.S. Department of Agriculture, Forest Service: 406-416.

Beck, D.E., and Della-Bianca, L. 1981. Yellow-poplar: Characteristics and management. Agric. Handb. 583. Asheville, NC: U.S. Department of Agriculture, Forest Service, Southeastern Forest Experiment Station. 91 p.

Benzie, J.W. 1977. Manager’s handbook for red pine in the North Central States. Gen. Tech. Rep. NC-33. St. Paul, MN: U.S. Department of Agriculture, Forest Service, North Central Forest Experiment Station. 22 p.

Braiewa, M.A., Brown, J.H., Jr., and Gould, W.P. 1985. Biomass and cordwood production of red maple stands in Rhode Island. Journal of Forestry. 83(11): 683-685.

Braun, E.L. 1961. The woody plants of Ohio. Columbus, OH: Ohio State University Press. 362 p.

Brinkman, K.A.; Roe, E.I. 1975. Quaking aspen: silvics and management in the Lake States. Agric. Handb. 486. Washington, DC: U.S. Department of Agriculture, Forest Service. 52 p.

Carey, A.B. 1983. Cavities in trees in hardwood forests. In: Davis, Jerry W.; Goodwin, Gregory A.; Ockenfeis, Richard A., technical coordinators. Snag habitat management: proceedings of the symposium; 1983 June 7-9; Flagstaff, AZ. Gen. Tech. Rep. RM-99. Fort Collins, CO: U.S. Department of Agriculture, Forest Service, Rocky Mountain Forest and Range Experiment Station: 167-184.

Carey, A.B., and Gill, J.D. 1980. Firewood and wildlife. Res. Note 299. Broomall, PA: U.S. Department of Agriculture, Forest Service, Northeastern Forest Experiment Station. 5 p.

Chapman, W.K., and Bessette, A.E. 1990. Trees and shrubs of the Adirondacks. Utica, NY: North Country Books, Inc. 131 p.

Clewell, A.F. 1985. Guide to the vascular plants of the Florida Panhandle. Tallahassee, FL: Florida State University Press. 605 p.

Collingwood, G.H. 1937. Knowing your trees. Washington, DC: The American Forestry Association. 213 p.

Crow, T. R. 1990. Tilia americana L. American basswood. In: Burns, Russell M.; Honkala, Barbara H., technical coordinators. Silvics of North America. Volume 2. Hardwoods. Agric. Handb. 654. Washington, DC: U.S. Department of Agriculture, Forest Service: 784-791.

Curtis, J.T. 1959. The vegetation of Wisconsin. Madison, WI: The University of Wisconsin Press. 657 p.

DeByle, N.V. 1985. Water and watershed. In: DeByle, Norbert V.; Winokur, Robert P., eds. Aspen: ecology and management in the western United States. Gen. Tech. Rep. RM-119. Fort Collins, CO: U.S. Department of Agriculture, Forest Service, Rocky Mountain Forest and Range Experiment Station: 153-160.

DeGraaf, R.M., and Shigo, A.L. 1985. Managing cavity trees for wildlife in the Northeast. Gen. Tech. Rep. NE-101. Broomall, PA: U.S. Department of Agriculture, Forest Service, Northeastern Forest Experiment Station. 21 p.

Dugger, K.M., and Fredrickson, L.H. 1992. Life history and habitat needs of the wood duck. Fish and Wildlife Leaflet 13.1.6. Waterfowl Management Handbook. Washington, DC: U.S. Department of the Interior, Fish and Wildlife Service. 8 p.

Duncan, W.H., and Duncan, M.B. 1987. The Smithsonian guide to seaside plants of the Gulf and Atlantic Coasts from Louisiana to Massachusetts, exclusive of lower peninsular Florida. Washington, DC: Smithsonian Institution Press. 409 p.

Duncan, W.H., and Duncan, M.B. 1988. Trees of the southeastern United States. Athens, GA: The University of Georgia Press. 322 p.

Eyre, F.H., and Zehngraff, P. 1948. Red pine management in Minnesota. Circ. No. 778. Washington, DC: U.S. Department of Agriculture. 70 p.

Farrar, J.L. 1995. Trees of the northern United States and Canada. Ames, IA: Blackwell Publishing. 502 p.

Frank, R. M. 1990. Abies balsamea (L.) Mill. balsam fir. In: Burns, Russell M.; Honkala, Barbara H., technical coordinators. Silvics of North America. Volume 1. Conifers. Agric. Handb. 654. Washington, DC: U.S. Department of Agriculture, Forest Service: 26-35.

Gansner, D.A., and Widmann, R.H. 1990. Enough white ash for wooden bats? Northern Logger & Timber Processor. 38(10): 32-33.

Gilmore, M.R. 1919. Uses of plants by the Indians of the Missouri River region. 33rd Annual Report. Washington, DC: Bureau of American Ethnology. 154 p.

Godfrey, R. K. 1988. Trees, shrubs, and woody vines of northern Florida and adjacent Georgia and Alabama. Athens, GA: The University of Georgia Press. 734 p

Godman, R.M., and Tubbs, C.H. 1973. Establishing even-age northern hardwood regeneration by the shelterwood method--a preliminary guide. Res. Pap. NC-99. St. Paul, MI: U.S. Department of Agriculture, Forest Service, North Central Forest Experiment Station. 9 p

Graney, D.L. 1990. Carya ovata (Mill.) K. Koch shagbark hickory. In: Burns, Russell M.; Honkala, Barbara H., technical coordinators. Silvics of North America. Volume 2. Hardwoods. Agric. Handb. 654. Washington, DC: U.S. Department of Agriculture, Forest Service: 219-225.

Haag, C.L., Johnson, J.E., and Erdmann, G.G. 1989. Rooting depths of red maple (Acer rubrum L.) on various sites in the Lake States. NC-347. St. Paul, MN: U.S. Department of Agriculture, Forest Service, North Central Forest Experiment Station. 3 p.

Hardin, K.I., and Evans, K.E. 1977. Cavity nesting bird habitat in the oak-hickory forests--a review. Gen. Tech. Rep. NC-30. St. Paul, MN: U.S. Department of Agriculture, Forest Service, North Central Forest Experiment Station. 23 p.

Hardt, R.A., and Forman, R.T.T. 1989. Boundary form effects on woody colonization of reclaimed surface mines. Ecology. 70(5): 1252-1260.

Harlow, W.M., Harrar, E.S., and White, F. M. 1979. Textbook of dendrology. 6th ed. New York: McGraw-Hill, Inc. 510 p.

He, H.S., Mladenoff, D.J., and Gustafson, E.J. 2002. Study of landscape change under forest harvesting and climate warming-induced fire disturbance. Forest Ecology and Management. 155: 257-270.

Holloway, P.S., and Alexander, G. 1990. Ethnobotany of the Fort Yukon region, Alaska. Economic Botany. 44(2): 214-225.

Hosie, R.C. 1969. Native trees of Canada. 7th ed. Ottawa, ON: Canadian Forestry Service, Department of Fisheries and Forestry. 380 p.

Johnson, C.W., Brown, T.C., and Timmons, M.L. 1985. Esthetics and landscaping. In: DeByle, Norbert V.; Winokur, Robert P., eds. Aspen: ecology and management in the western United States. Gen. Tech. Rep. RM-119. Fort Collins, CO: U.S. Department of Agriculture, Forest Service, Rocky Mountain Forest and Range Experiment Station: 185-188.

Johnson, P.S. 1990. Quercus coccinea Muenchh. scarlet oak. In: Burns, Russell M.; Honkala, Barbara H., tech. coords. Silvics of North America. Vol. 2, Hardwoods. Agric. Handb. 654. Washington, DC: U.S. Department of Agriculture, Forest Service: 625-630.

Johnston, W.F. 1990. Thuja occidentalis L. northern white-cedar. In: Burns, Russell M.; Honkala, Barbara H., technical coordinators. Silvics of North America. Volume 1. Conifers. Agric. Handb. 654. Washington, DC: U.S. Department of Agriculture, Forest Service: 580-589.

Kriebel, H. B.; Gabriel, W. J. 1969. Genetics of sugar maple. Res. Pap. WO-7. Washington, DC: U.S. Department of Agriculture, Forest Service.17 p

Laidly, P.R. 1990. Populus grandidentata Michx. bigtooth aspen. In: Burns, Russell M.; Honkala, Barbara H., tech. coords. Silvics of North America: Volume 2. Hardwoods. Agric. Handb. 654. Washington, DC: U.S. Department of Agriculture, Forest Service: 544-550.

Lamson, N.I. 1983. Precommercial thinning increases diameter growth of Appalachian hardwood stump sprouts. Southern Journal of Applied Forestry. 7(2): 93-97.

Lanasa, M. 1989. Northern white-cedar management and whitetail deer habitat. In: Proceedings of the National Silviculture Workshop: Silviculture for all resources; 1987 May 11-14; Sacramento, CA. Washington, DC: U.S. Department of Agriculture, Forest Service, Timber Management: 19-24.

Lees, J.C. 1981. Three generations of red maple stump sprouts. Information Report M-X. Fredericton, New Brunswick: Maritimes Forest Research Centre, Canadian Forestry Service, Environment Canada. 9 p.

Little, S., Moorhead, G.R., and Somes, H.A. 1958. Forestry and deer in the Pine Region of New Jersey. Station Pap. No. 109. Upper Darby, PA: U.S. Department of Agriculture, Forest Service, Northeastern Forest Experiment Station. 33 p.

Maeglin, R. R. 1974. The effect of site quality and growth rate on the anatomy and utilization potential of northern red oak. In: Proceedings of the second annual hardwood symposium; 1974 May 2 - May 4; [Location of conference unknown]. [Place of publication unknown]. Hardwood Research Council: 191-205.

Marquis, D.A. 1990. Prunus serotina Ehrh. black cherry. In: Burns, Russell M.; Honkala, Barbara H., technical coordinators. Silvics of North America. Volume 2. Hardwoods. Agric. Handb. 654. Washington, DC: U.S. Department of Agriculture, Forest Service: 594-604.

Martin, A.C., Zim, H.S., and Nelson, A.L. 1951. American wildlife and plants. New York: McGraw-Hill Book Company, Inc. 500 p.

McQuilkin, R.A. 1990. Quercus prinus L. chestnut oak. In: Burns, Russell M.; Honkala, Barbara H., tech. coords. Silvics of North America. Vol. 2. Hardwoods. Agric. Handb. 654. Washington, DC: U.S. Department of Agriculture, Forest Service: 726 p.

Michael, E.D. 1988. Effects of white-tailed deer on Appalachian hardwood regeneration. In: Smith, H. Clay; Perkey, Arlyn W.; Kidd, William E., Jr., eds. Guidelines for regenerating Appalachian hardwood stands: Workshop proceedings; 1988 May 24-26; Morgantown, WV. SAF Publ. 88-03. Morgantown, WV: West Virginia University Books: 89-96.

Millers, I., Shriner, D.S., and Rizzo, D. 1989. History of hardwood decline in the eastern United States. Gen. Tech. Rep. NE-126. Bromall, PA: U.S. Department of Agriculture, Forest Service, Northeastern Forest Experiment Station. 75 p.

Monk, C.D. 1968. Successional and environmental relationships of the forest vegetation of north central Florida. American Midland Naturalist. 79(2): 441-457.

Morden-Moore, A.L., and Willson, M.F. 1982. On the ecological significance of fruit color in Prunus serotina and Rubus occidentalis: field experiments. Canadian Journal of Botany. 60: 1554-1560.

Morley, P.M., and Balatinecz, J.J. 1993. Poplar utilization in Canada: past, present and future. Forestry Chronicle. 69(1): 46-52.

Moser, H.C. 1971. Manufacture of oak furniture, cabinets, and panels. In: White, D. E.; Roach, B. A., co-chairmen. Oak symposium proceedings; 1971 August 16-20; Morgantown, WV. Upper Darby, PA: U.S. Department of Agriculture, Forest Service, Northeastern Forest Experiment Station: 100-102.

Newton, M., Cole, E.C., Lautenschlager, R. A.; [and others]. 1989. Browse availability after conifer release in Maine's spruce-fir forests. Journal of Wildlife Management. 53(3): 643-649.

Nowacki, G.J., and Abrams, M.D. 1992. Community, edaphic, and historical analysis of mixed oak forests of the Ridge and Valley Province in central Pennsylvania. Canadian Journal of Forest Research. 22: 790-800.

Olson, D.F., Jr. 1974. Quercus L. oak. In: Schopmeyer, C. S., ed. Seeds of woody plants in the United States. Agric. Handb. 450. Washington, DC: U.S. Department of Agriculture, Forest Service: 692-703.

Ontario Department of Lands and Forests. 1953. Forest tree planting. 2ded. Bull. No. R 1. Toronto, Canada: Ontario Department of Lands and Forests, Division of Reforestation. 68 p.

Peek, J. M. 1974. A review of moose food habits studies in North America. Le Naturaliste Canadien. 101: 195-215.

Pekins, P.J., and Mautz, W.W. 1988. Digestibility and nutritional value of autumn diets of deer. Journal of Wildlife Management. 52(2): 328-332.

Perala, D.A., and Carpenter, E.M. 1985. Aspen: An American wood. FS-217. Washington, DC: U.S. Department of Agriculture, Forest Service. 8 p.

Rudolf, P.O. 1990. Pinus resinosa Ait. red pine. In: Burns, Russell M.; Honkala, Barbara H., technical coordinators. Silvics of North America. Volume 1. Conifers. Agric. Handb. 654. Washington, DC: U.S. Department of Agriculture, Forest Service: 442-455.

Safford, L. O.; Bjorkbom, J.C.; and Zasada, J.C. 1990. Betula papyrifera Marsh. paper birch. In: Burns, Russell M.; Honkala, Barbara H., technical coordinators. Silvics of North America. Vol. 2. Hardwoods. Agric. Handb. 654. Washington, DC: U.S. Department of Agriculture, Forest Service: 158-171.

Sander, I.L. 1990. Quercus rubra L. northern red oak. In: Burns, Russell M.; Honkala, Barbara H., technical coordinators. Silvics of North America. Volume 2. Hardwoods. Agric. Handb. 654. Washington, DC: U.S. Department of Agriculture, Forest Service: 727-733.

Schlesinger, R.C. 1990. Fraxinus americana L. white ash. In: Burns, Russell M.; Honkala, Barbara H., technical coordinators. Silvics of North America. Vol. 2. Hardwoods. Agric. Handb. 654. Washington, DC: U.S. Department of Agriculture, Forest Service: 333-338.

Sims, R.A., Kershaw, H.M., and Wickware, G.M. 1990. The autecology of major tree species in the north central region of Ontario. COFRDA(Canada-Ontario Forest Resources Development Agreement) Report 3302; NWOFTDU (Northwestern Ontario Forest Technology Development Unit) Technical Report 48. Ottawa: Forestry Canada, Ontario Region; Thunder Bay, ON: Ontario Ministry of Natural Resources, Northwestern Ontario Forest Technology Development Unit. 126 p

Smalley, G.W. 1990. Carya glabra (Mill.) Sweet pignut hickory. In: Burns, Russell M.; Honkala, Barbara H., technical coordinators. Silvics of North America. Vol. 2. Hardwoods. Agric. Handb. 654.

Sork, V.L., Stacey, P., and Averett, J.E. 1983. Utilization of red oak acorns in non-bumper crop year. Oecologia. 59: 49-53.

Stephens, H. A. 1973. Woody plants of the North Central Plains. Lawrence, KS: The University Press of Kansas. 530 p.

Stephens, H. A. 1980. Poisonous plants of the central United States. Lawrence, KS: The Regents Press of Kansas. 165 p.

Stormer, F.A., and Bauer, W.A. 1980. Summer forage use by tame deer in northern Michigan. Journal of Wildlife Management. 44(1): 98-106.

Telfer, E.S. 1972. Browse selection by deer and hares. Journal of Wildlife Management. 36(4): 1344-1349.

Tew, R.K. 1970. Seasonal variation in the nutrient content of aspen foliage. Journal of Wildlife Management. 34(2): 475-478.

Torbert, J. L.; Tuladhar, A. R.; Burger, J. A.; Bell, J. C. 1988. Minesoil property effects on the height of ten-year-old white pine. Journal of Environmental Quality. 17(2): 189-192.

Van Dersal, W.R. 1938. Native woody plants of the United States, their erosion-control and wildlife values. Washington, DC: U.S. Department of Agriculture. 362 p.

Van Dersal, W.R. 1940. Utilization of oaks by birds and mammals. Journal of Wildlife Management. 4(4): 404-428.

Vines, R.A. 1960. Trees, shrubs, and woody vines of the Southwest. Austin, TX: University of Texas Press. 1104 p.

Vogel, W.G. 1981. A guide for revegetating coal mine soils in the eastern United States. Gen. Tech. Rep. NE-68. Broomall, PA: U.S. Department of Agriculture, Forest Service, Northeastern Forest Experiment Station. 190 p.

Watson, L.E., Parker, R.W., and Polster, D. F. 1980. Manual of plant species suitability for reclamation in Alberta. Vol. 2. Forbs, shrubs and trees. Edmonton, AB: Land Conservation and Reclamation Council. 537 p.

Wendel, G.W., and Smith, H.C. 1990. Pinus strobus L. eastern white pine. In: Burns, Russell M.; Honkala, Barbara H., technical coordinators. Silvics of North America. Volume 1. Conifers. Agric. Handb. 654. Washington, DC: U.S. Department of Agriculture, Forest Service: 476-488.

Youngquist, J.A., and Spelter, H. 1990. Aspen wood products utilization: impact of the Lake States composites industry. In: Adams, Roy D., ed. Aspen symposium '89: Proceedings of symposium; 1989 July 25-27; Duluth, MN. Gen. Tech. Rep. NC-140. St. Paul, MN: U.S. Department of Agriculture, Forest Service, North Central Forest Experiment Station: 91-102.
